# Consumer-friendly food allergen detection: moving towards smartphone-based immunoassays

**DOI:** 10.1007/s00216-018-0989-7

**Published:** 2018-03-26

**Authors:** Georgina M. S. Ross, Monique G. E. G. Bremer, Michel W. F. Nielen

**Affiliations:** 1 RIKILT, Wageningen University and Research, P.O Box 230, 6700 AE Wageningen, The Netherlands; 2 Laboratory of Organic Chemistry, Wageningen University, Helix Building 124, Stippeng 4, 6708 WE Wageningen, The Netherlands

**Keywords:** Food allergen, Immunoassay, Smartphone, Consumer, Multiplex, Citizen science

## Abstract

In this critical review, we provide a comprehensive overview of immunochemical food allergen assays and detectors in the context of their user-friendliness, through their connection to smartphones. Smartphone-based analysis is centered around citizen science, putting analysis into the hands of the consumer. Food allergies represent a significant worldwide health concern and consumers should be able to analyze their foods, whenever and wherever they are, for allergen presence. Owing to the need for a scientific background, traditional laboratory-based detection methods are generally unsuitable for the consumer. Therefore, it is important to develop simple, safe, and rapid assays that can be linked with smartphones as detectors to improve user accessibility. Smartphones make excellent detection systems because of their cameras, embedded flash functions, portability, connectivity, and affordability. Therefore, this review has summarized traditional laboratory-based methods for food allergen detection such as enzyme-linked-immunosorbent assay, flow cytometry, and surface plasmon resonance, and the potential to modernize these methods by interfacing them with a smartphone readout system, based on the aforementioned smartphone characteristics. This is the first review focusing on smartphone-based food-allergen detection methods designed with the intention of being consumer-friendly.

**Electronic supplementary material:**

The online version of this article (10.1007/s00216-018-0989-7) contains supplementary material, which is available to authorized users.

**Graphical abstract:**
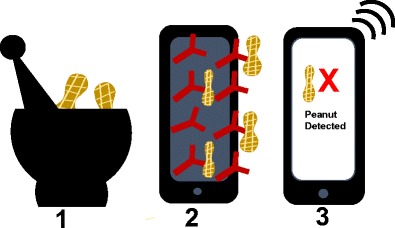
A smartphone-based food allergen detection system in three easy steps (1) sample preparation, (2) allergen detection on a smartphone using antibodies, which then transmits the data wirelessly, (3) analytical results sent straight to smartphone

## Introduction

An allergen is a protein capable of eliciting an immune response in sensitized individuals. Food allergies represent a significant international health problem. Worldwide, allergies toward foods affect 2% of the adult population and 5%–8% of the children population [1, 2]. There are many existing methods for food allergen detection, which can be split into two general categories: protein-based and DNA-based detection. For a general and in-depth explanation on all in-vivo and in-vitro allergen assays, the review by Poms et al. can be referred to [3]. General and quantitative methods for allergen detection have been reviewed by Kirsch et al. and Walker et al. [2, 4]. Additionally, an overview on commercially available rapid immuno-analytical allergen detectors has been presented by Schubert-Ulrich et al. [5]. All immunochemical and DNA-based methods were reviewed by Monaci and Visconti and by Slowianek [6, 7]. Further discussion into allergen detection methods with a particular focus on proteomic mass-spectrometry has been given by Prado et al. [8]. The most recently published food allergen review [9] focused on the use of biosensors for detection, so only limited attention will be paid to them in this review.

Although analytical methods such as mass spectrometry can provide a wealth of information when used complementarily with immuno-methods; current allergen analysis trends are moving away from lab methods and toward point-of-care diagnostics (PoC) and a citizen science approach [10]. Point-of-care diagnostics allow instant on-site testing for food allergens by individuals, whilst citizen science centers around consumer-friendly devices that allow users to carry out their own PoC allergen analysis. It is of particular importance that food allergen detection devices are consumer-friendly as allergic individuals will need to carry out testing at home or in restaurants prior to eating. Many allergic individuals suffer from more than one food allergy, due to cross-reactivity, where antibodies against one allergen recognize a structurally related epitope of another similar allergen [11]. Owing to allergens being cross-reactive, it is necessary to develop multiplex devices that can detect a range of allergens within a single sample, saving time and money and making sure that the consumers are confident that their food does not contain any undesired allergens. For the purpose of this review, a consumer can be considered as the end-user of the assay/detector, and thus the terms consumer and user are used synonymously. The authors define consumer friendly to mean that any adult of average intelligence would be able to perform the assay safely and effectively with minimal instruction. One way of making allergen testing more user-friendly is to link the assays with a smart-detector such as: a smartphone, tablet, or wearable device. Although some of the existing allergen assay formats are simple to perform, linking these tests to a smart detector will make them more accessible for the general public. As the majority of the population already owns a smartphone, with the number rising, smartphones represent a source of analytical equipment that can reach even the most desolate areas of the globe, making them ideal for sensors [12].

Smartphones are ideal to use as detector systems because of their powerful internal computers, optical sensors, global positioning systems (GPS), and most importantly, their ability to connect to the internet, through Bluetooth and WiFi [13–15]. Connectivity is a key benefit of smartphones as results can instantaneously be uploaded to Cloud databases and results can be disseminated as spatio-temporal maps across the globe [16]. Since their development in 1992 and first use as analytical devices in 2008, smartphones have already been used as sensors, for light microscopy, single-molecule microscopy, cell imaging, bacteria detection, colorimetric detection, enzyme linked immunosorbent assay (ELISA), and lateral flow immunoassays (LFIA), which exemplifies their capabilities as detectors in rapid diagnostics [12, 17–26] . For an in-depth review into all existing smartphone-based diagnostic devices, Quesada-Gonzalez and Merkoçi can be referred to [27]. For a more focused review concerning biosensors and bioelectronics on a smartphone see Zhang and Liu [28]. General approaches to smartphone-based food diagnostics have been recently reviewed by Rateni et al. [29] and Choi [12], which addressed the necessity and market-gap for user-friendly food detection. This is particularly important in the field of food allergen analysis where detection methods must be consumer-friendly so that the allergic individuals can apply analysis themselves in the comfort of their home and/or at a restaurant. The present review specifically focuses on how successful lab-based methods can be based on smartphones to enable consumer-friendly allergen detection.

Up until now, the literature has lacked specific focus on consumer-friendly food allergen detection devices. To that end, literature has been reviewed from the period of 2002 to the end of 2017 using the SciFinder, Scholar, Scopus, and Web of Science databases and key words such as: food allergen, detection, smartphone or cell phone, multiplex, lateral flow, immunoassay, cross-flow, microfluidics, strip reader, and ELISA. Section 1 of the review will provide a general background of food allergens and the legislations that control the labelling procedures. The study will then discuss the concept of consumer friendliness in Section 2. In Section 3 there will be focus on traditional laboratory-based methods for food allergen analysis and how these methods could improve their consumer friendliness through coupling to a smartphone as a detector. Section 4 will discuss assays/devices that have been designed with the intention of being consumer-friendly, including commercial consumer-friendly allergen detectors. Finally, the conclusion will summarize the findings of the review.

## Background on food allergens

### Types of food allergens

Food allergies can be debilitating, and food requires proper monitoring to ensure sensitized individuals are not exposed to allergens. Symptoms of food allergy can include: itching, diarrhoea, stomach pains, eczema, shortness of breath as well as more significant effects such as loss of consciousness and anaphylactic shock, which can be fatal [30]. The prevalence of food allergies is increasing, but awareness of allergies is growing even faster with dedicated events such as ‘Food Allergy Awareness Week’ in the USA [31]. The Codex Alimentarius Standard listed eight allergens with international variants, which require mandatory labelling [32]. These are referred to as the Big 8 and consist of: peanuts, tree-nuts, milk, eggs, fish, crustacean, soya, and wheat [33]. Wheat contains a variety of proteins that have been implicated as allergens (see Table 1). In addition to wheat allergy, other wheat-related disorders include the autoimmune disorder, celiac disease. Celiac disease is triggered by gluten, a protein mixture of prolamins and glutelins, which can be found in wheat, rye, barley, and their cross-breeds [58]. Allergic reactions are provoked by many different proteins within the allergenic foods. Those allergenic proteins which have been repeatedly referenced in the literature and databases (e.g., allergen.org) as causing an allergic reaction in the majority of sensitised individuals are described in Table 1 below.

**Table 1:** The main allergenic proteins in foods within the ‘Big 8’ plus ‘gluten’

Food	Major allergenic protein	Reference
Cow’s Milk	B-lactoglobulin (Bos d 5)	[34]
	Casein (Bos d 8)	[35]
	α-lactalbumin (Bos d 4)	[36]
Egg	Ovomucoid (Gal d 1)	[37]
	Ovalbumin (Gal d 2)	[37]
	Ovotransferrin (Gal d 3)	[38]
	Lysozyme (Gal d 4)	[39]
	α-livetin (Gal d 5)	[40]
Crustacean	Tropomyosin (Pen a 1)	[41]
Fish	Β-parvalbumin (Lep w 1; Pon 1 4; Pon 1 7; Seb m 1; Xip g 1)	[42]
Peanut	Ara h1	[43]
	Ara h2	[44]
	Ara h3	[45]
	Arah h4-9	[46]
Tree nuts		
Hazelnut	Cor a 1; Cor a 2; Cor a 8; Cor a 9; Cor a 11; Cor a 12; Cor a 13; Cor a 14	[47]
Brazil nut	Ber e 1; Ber e 2	[48]
Cashew	Ana o 1; Ana o 2; Ana o 3	[49]
Almond	Pru du 3; Pru du 4; Pru du 5; Pru du 6	[50]
Walnut (Black)	Jug n 1; Jug n 2; Jug n 4	[42]
Walnut (English)	Jug r 1-6	[42]
Pecan	Car i 1; Car i 2; Car i 4	[42]
Pistachio	Pis v 1; Pis v 2; Pis v 3; Pis v 4; Pis v 5	[42]
Soybean	Gly m Bd 30K	[51]
	Gly m Bd 60K	[52]
	Gly m Bd 28K	[52]
Wheat	Tri a 12	[53, 54]
	Tri a 14	[53, 55]
	Tri a 18	[53, 56]
	Tri a 25	[53, 56, 57]
Gluten*	Gluten (Tri a 26 & Tri a 36)	[42, 53]
	Gliadin (Tri a 19 & Tri a 20)	[42, 53]

*Although not an allergen, gluten has been included in this table to show the toxic portion of the protein responsible for gluten’s autoimmune effects_._

Allergenic proteins can result in hypersensitivity of the immune system, arbitrated by allergen-specific immunoglobulin E (IgE) (type I allergies); but allergies can also be cell-mediated (non-IgE) (type II allergies) [9, 59]. Disruption of the structure of allergens by food processing can lead to an increase or decrease in their immunogenicity, altering how an allergic individual might react to the protein [60]. The modification of allergenic proteins is dependent on the processing procedure applied. For example, by hydrolyzing or thermally treating an allergen, the structure is altered, which can result in either a reduction in immunogenicity of the allergen, or the formation of a neo-allergen. The method used for processing a food will affect the extractability of the allergens from their matrix [61]. When extracting gluten, for example, it is crucial to have a homogenized sample so that particulates can be extracted. As ethanol-based extractions result in the incomplete extraction of gluten, it is desirable to use a cocktail extraction solution that contains a reducing agent and alcohol, which is capable of extracting monomeric and polymeric proteins from gluten [62–64]. Extraction procedures have been a detriment in the past, where hazardous and environmentally damaging extraction solutions such as 2-mercaptoethanol (2-ME) have been applied in food allergen extraction [65]. In order to step toward consumer-friendliness it is necessary to have extraction buffers that are safe to use and easy to dispose of. Many traditional allergen analysis methods use environmentally harmful reagents, which contain additives that improve allergen solubility/extractability and reduce background interference from the food matrix [66]. It is desirable to use eco-friendly extraction buffers, but these must first be compared and validated against traditional buffers to ensure that they are as effective in allergen extraction.

All assays and detectors need to be effectively validated by standardized procedures. Certified reference materials in raw and processed foods need to be developed for food allergens as well as reference methods for allergen analysis [67, 68]. Current lack of standardized reference materials for allergens in foods means that there is a lack of consistency between different allergen detection methods as each test kit is calibrated in a different way. Reference materials are critical for quality assurance of allergen detection methods, but their production is complicated in food allergen analysis owing to the changes in allergen protein structure during food processing procedures [6]. When standardized reference materials are developed, they should be based on a whole protein extract as allergens are a mixture of non-defined proteins in complex matrices [69]. Having a set of standards for allergen testing devices will ensure that effective and smart detection devices can be created, validated, and benchmarked against each other, allowing consumer science to be achieved by providing individuals with personalized smart-detection platforms for food allergens.

### Worldwide legislation and mandatory labelling

Worldwide, dietary differences and the Big 8 influence which allergens require mandatory labelling. Some countries include additional mandatory and recommended allergens for labelling depending on the staple diet of that particular country [70]. Despite worldwide communication, significant variance exists in different countries’ regulatory labelling framework. This can be problematic due to the high percentage of international food trade and individual people’s travelling patterns [71].

The European Commission (EC) produced legislation in 2003 (Directive 2003/89/EC) covering a list of 14 allergens that require mandatory labelling; the legislation is commonly referred to as the “allergen-labelling-directive” [72]. If a manufacturer uses any of the allergens listed, it must be stated, with clear labelling, on the packaging [73]. This is a crucial amendment, as labelling of the presence of allergenic ingredients is currently the only way allergic individuals can effectively maintain strict avoidance diets [74]. Proper labelling of allergens is crucial as it informs consumers what products are safe to eat. In 2014, the EU Regulation amendment 1169/2011 came officially into effect. This amendment stipulated that even non-prepackaged foods require allergen labelling, meaning in practice that all food retailers must provide allergen information [72, 75]. Food manufacturers and retailers are responsible for the proper labelling of their products; when an allergen has been labelled, it then becomes the consumer’s responsibility to avoid this food [68]. As a large amount of food allergic reactions happen to individuals when they are abroad, it is vital that consumers are aware of the differences in which allergens require labelling in other countries (see Electronic Supplementary Material (ESM) Table S1). However, it is undeclared food allergens that are accidentally introduced into non-allergenic foods, through cross-contamination, that pose the biggest risk to the consumer [76]. The EU does not currently provide guidance on labelling for allergens that may have unintentionally been introduced into the product via shared facilities [72].

### Precautionary labelling and thresholds

The EU has a zero tolerance policy for allergen labelling, and any foods listed in the legislation (see ESM Table S1) must be stated on the food packaging when they are used as ingredients or processing aids in the food. However, the EU has no obligation to label any allergens that are not part of the recipe and may have accidentally been introduced by cross-contamination [67]. Some countries have set threshold levels, and any food containing allergens above those levels require labelling. For example, in Japan, any foods containing any of the legislated allergens (see ESM Table S1) above 10 ppm must be declared on the packaging, meaning that the majority of the allergic population are protected from exposure [70]. However, due to individual differences in sensitivities to allergens, having such a low labelling threshold may further restrict the diet of individuals who are less sensitive to those allergens. Switzerland has taken an alternative approach, not mandating allergen labelling for any product containing less than 1000 ppm of allergen [77]. The Swiss approach can be detrimental to the allergic individual, with many people experiencing allergic reactions at levels far lower than 1 g/kg for particular allergens [78]. The Swiss allergen labelling legislation illuminates the requirement for consumers to be able to test their own foods for allergen presence so that they do not have to solely rely on labelling legislations.

In addition, it is also common practice for food manufacturers to include precautionary allergen labelling (PAL) on their foods for protection against unintentional presence of allergens. There is a lack of consistency in the wording of PAL, which can be confusing to the allergic consumer and reduces the consumer’s ability to make informed food choices [68]. Labels such as “may contain nuts” are used if there is any risk the product may have come into contact with an allergen [77]. Food manufacturing companies have highlighted their desire for standardised PAL on food packaging to avoid misinterpretation [79]. Although advisory labelling is well-intentioned, excessive use of warnings can lead to individuals taking risks with what they eat by ignoring the labels [80–82]. Currently, most countries’ PAL is not on threshold-based criteria, and manufacturers include labels for any potential allergen.

There is an evident requirement for threshold-based action levels, to properly assess the risk of an unintentional allergen being introduced to a food, and to establish when and where advisory labelling is necessary and beneficial to the allergic consumer. These action levels should be science-based. Clinical information regarding minimum eliciting doses has been translated into lowest-observed adverse effect levels (LOAEL) and no observed adverse effect levels (NOAEL) [78, 83, 84]. Developing effective thresholds using LOAELs is a safety assessment-based approach that protects the majority of the allergic population. The Allergen Bureau of Australia and New Zealand (ABA) is a global leader in regulation of labelling and has already established voluntary labelling thresholds for the major allergens, based on LOAELs, which protect 95% of allergic population from severe reactions [82, 85]. Voluntary Incidental Trace Allergen Labelling (VITAL) aims to limit the use of excessive, unnecessary PAL in foods; and has also incorporated reference dose information into the LOAEL action levels for allergen labelling [82, 86]. The reference dose in VITAL is defined as milligrams of total protein from an allergenic food from which only the most sensitive individual would be likely to experience an adverse reaction [87]. If the individual reference dose is exceeded in an unlabelled food, VITAL recommends precautionary labelling [67]. In 2011, a scientific expert committee including the food allergy research and resource program (FARRP), revised VITAL to develop VITAL 2.0, which uses action levels based on reference doses [88]. The action levels provide a clear indication on when “may contain” labelling should be applied. Despite Australia and New Zealand being at the forefront of allergen labelling regulation, further implementation and standardization in PAL is required [85].

Regardless of dedicated labelling procedures, presence of undeclared allergens still remains the greatest cause for food-based recalls globally [31, 89]. Large scale recalls can have a significant socio-economic burden on a country [90]. The Rapid Alert System for Food and Feed (RASFF) is a European food safety risk assessment system that has experienced an increased volume of notifications regarding undeclared allergens in recent years [91]. When an allergen has been mislabelled, it must be reported to the competent authority as well as recalled in the notifying country and then RASFF issues an alert informing that the product contains a mislabelled allergen [92]. It is an option to notify RASFF about allergens that may have been unintentionally introduced into a product by cross-contamination; however, this is not mandatory as it is not regulated by the EU. Risk communication is expected within the food industry, but it is not mandatory, so providing the industry with sensitive tests that can detect allergens at concentrations as low as reasonably achievable (ALARA) is the best way to ensure that unintentional allergen presence in food is monitored. In order for consumers to be entirely confident that their food is free from allergens, it is necessary to manufacture easy to use assays to detect unwanted allergen presence so that consumers do not have to rely on recall or notification data to maintain their avoidance diets [93, 94]. A consumer-friendly allergen test that can be based on a smart-detector could provide consistent, essential information for the allergic individual, regardless of the quality of product labelling.

### Criteria for consumer-friendliness

As the world moves towards personalized testing and diagnosis, the need for user-friendly devices becomes more apparent. Whilst many products claim to be ‘for the consumer’, in reality only a low percentage of these devices actually are. It is useful to consider the parameters that make an assay usable for the general population. Recently, stakeholder guidance into the development of consumer-orientated allergen analytical devices has highlighted the need for standardization of instructions for assay use and for transparency in validation procedures in consumer assays [95]. For a truly user-friendly assay, the majority of the adult population should be able to perform it successfully; using the device should be self-explanatory or require minimal instruction. When linking an assay to a smartphone app it is possible to include safety information and instructions for application within the app, limiting the need for an instruction manual. Alongside being easy-to-use, the assay should be safe and not contain toxic chemicals; it should also not be able to stain the user/damage clothing and therefore should not require the use of personal protective equipment (PPE). There should be no toxic waste produced, and preferably the assay should be environmentally friendly and recyclable; there should be instructions on how to dispose any waste that does come from the assay [95].

The assay/detector should require minimal external equipment. By having to use scientific equipment such as precision pipettes and centrifuges, the manufacturer introduces the need for further training/explanation to negate human error. In addition, requiring basic laboratory skills (such as pipetting), prevents individuals with no scientific background from being able to use the device. External equipment increases the overall cost of the assay, and affordability is a prerequisite for user-friendly assays. Pre-containing reagents within the assay eliminates pipetting steps and allows waste to be minimized and cost reduced. As the consumer cannot rely solely on the visual readout of a screening assay, another major cost in many assays is the requirement for a specialized detector/reader [95]. Next-generation citizen science detectors such as smartphones reduce cost significantly, as most people already own at least one smartphone. Often the assay can be performed with relative ease (e.g., LFIA) but it is the result interpretation, such as differentiating between lighter and darker lines, which is difficult for the consumer and can be negatively affected by personal bias. In general, LFIA readers are expensive and are not something that consumers would own and carry around with them, whereas smartphones are universally present across the globe. The smartphone as a readout system makes most assays more consumer-friendly as the majority of people are accustomed to using smartphone applications. A significant benefit of using the smartphone is that the results can be instantly uploaded to Cloud databases/sent to relevant stakeholders, which can be particularly useful for remote quality control. Conversely, it should be considered that when using a smartphone-based analytical device in a low resource setting, the wireless system may suffer with low connectivity, and so the smartphone application must be able to support asynchronous data transmission [12]. Linking an assay to a smartphone detector goes a long way in making the assay more portable. Portability means that the assay can be taken anywhere and applied under in-field conditions, such as in a restaurant.

Another key component of a user-friendly device is that it should provide results quickly. Consumers do not want to wait for extended periods for results, so rapid tests are desirable. The assay should provide results as quickly as reasonably possible without compromising the sensitivity or reliability of the test. The speed of an assay can be optimized by first carrying out detailed kinetics studies to select antibodies with rapid association rates and high affinities to the allergen of interest, for use in the assay. The reaction rate can also be increased by proper orientation of the antibodies, so that the relevant binding sites are directed away from the surface, where they can better interact with the targets. Assays can be further sped up by using internal microfluidics, which also limits the necessity for excessive sample handling/preparation as mixing can be achieved in the fluidics system. Microfluidics often increase the speed of the assay as mixing, pumping, and directional flow can be carried out at precise locations in the assay itself, limiting the need for operator interaction [96]. Proper mixing can also speed up the assay by increasing the rate of diffusion of the sample. The assay should not have significant cross-reactivity with different allergens, so that users can be certain that their results are correct. Proper characterization of antibodies ensures that the assay is selective for the target allergen. In addition to being selective, the assay should be sensitive and able to detect allergens at their LOAEL.

Multiplexing allows multiple allergens to be detected in a single sample, which is desirable, saving time and money in comparison with using several singleplex assays [97]. Furthermore, a proportion of the allergic population suffers from more than one allergy, due to cross-reactivity with similarly structured allergens, so it is attractive to test more than one allergen at a time [96–99]. An individual who suffers from multiple allergies should be able to test for all of them using a singular device. As allergens are structurally different proteins, they may require different extraction procedures; when testing for multiple allergens the extraction buffer will likely be a compromise between maximum extraction efficiency and the ability to co-extract different allergens from the matrix. Truly personalized allergen testing where consumers select the allergen panel they want included in the assay would come at an expense, but this could be lowered if companies start including more allergens in multiplex assays. The current proof-of-concept allergen multiplex assays are displayed in ESM Table S2.

It is critical for user-friendly assays to be reproducible so that the user is confident in the result. In order for this to be achieved, assays should be validated by intra- and inter-laboratory testing and benchmarked against successful commercial allergen assays. By proper validation, the reliability of the assay can be proved and consumer confidence can be attained. Popping et al. suggest that consumer devices should first go through single laboratory validation, followed by independent laboratory validation and proficiency testing in parallel, including being tested by untrained personnel/consumers [95]. It would improve the affordability of the assay if the assay were reusable such as when using a SPR chip; however, if the assay cannot be reused (LFIA) the smartphone attachment and app should be able to be reused for a number of cycles and the assay should be recyclable. The ideal device for consumers would therefore be: easy to use, safe, recyclable, affordable, a smartphone-based detector or other smart device with connectivity possibilities, portable, rapid, sensitive, multiplexed where appropriate, and properly validated and benchmarked.

## Methods for food allergen detection using a smartphone readout system

Immunochemical methods for allergen detection focus around the complementary interaction of an allergen-specific antibody and an allergen. An overview of commercial laboratory-based allergen assays is provided in ESM Table S3. Lab-based methods are highly sensitive, selective, and accurate. However, lab-based methods require trained personnel, scientific knowledge, and often expensive equipment. By linking traditional lab-based methods with a smartphone readout system, they become more user-accessible. A comparison of lab- and smartphone-based methods is given in ESM Table S4. The most popular optical approach to smartphone detectors is based on colorimetric reactions such as in LFIA or ELISA [28]. Colorimetric smartphone-based sensing conventionally relies on the phone’s complementary metal oxide semiconductor (CMOS) filters to assign red, green, blue (RGB) values to light. Therefore, smartphone-based sensors are able to detect changes in optical density or intensity of analyte–reagent complexes over a range of wavelengths [12]. The majority of the population has and is familiar with smartphones, so interfacing a scientific method with a simple smartphone app improves consumer friendliness.

### Lateral flow immunoassays

Lateral flow immunoassays (LFIA) are immuno-chromatographic test strips designed to be easy to use, as has been exemplified by their success as pregnancy tests [100]. Many food manufacturers utilize LFIA to test their clean-in-place (CIP) procedures and to ensure that their production lines are free from allergens. Cross-contamination can be monitored for instance using Lab-2-go, a user-friendly test toolkit developed by Zeulab (Zaragoza, Spain) to prove good manufacturing practice (GMP) [101]. The standard components of a LFIA are: the sample filter pad, the conjugate pad, the membrane, the absorption pad, and the test/control lines [102].

In a sandwich format LFIA, the conjugate pad contains a pre-sprayed antibody that is specific to the allergen of interest. This specific antibody is labelled with colored or fluorescent moieties. The test line contains an immobilized allergen-specific antibody, which binds to a different epitope on the allergen than the labelled antibody. The control line contains an antibody raised against the animal species of the labelled antibody. When a sample containing the target allergen is added to the sample pad, the target binds with the labelled antibody in the conjugate pad, forming a labelled complex. The labelled complex flows via capillary action, driven by the absorption pad, laterally up the membrane. When the test line is reached, the complex is captured by the immobilized allergen-specific antibody. The target analyte is sandwiched between the labelled and the captured antibodies, which results in the appearance of a colored line in the test region. The remaining labelled antibody binds with the immobilized anti-species antibody at the control line, resulting in the appearance of a second colored line in this region. In a sandwich assay, the color intensity of the test line is directly proportional to the concentration of the target allergen in the sample. Whilst the test line informs the user of the relative concentration of the allergen in the sample, the control line proves that the assay is functioning correctly.

#### *Multiplex dipstick tests*

Lateral flow immunoassays can also be multiplexed through the addition of multiple test lines. Each test line corresponds to the target analytes to be detected [103]. Detecting a range of allergens in a sample is attractive as it reduces analysis time and reagent waste, as multiple analytes can be assessed under the same conditions. Structures other than simple strip tests can also be applied in multiplexing. Fenton et al. have shown that two-dimensional shaping of capillary driven membrane assays into candelabra or other structures can improve the spatial discrimination of the assay [104]. Assays for different analytes can be positioned on separate arms of the device, which can be directly labelled to minimize user confusion. Currently, much of the attention of multiplex flow assays has been focused towards mycotoxin analysis [105]. It is expected that future research will focus on incorporating multiplex into the food allergen field in order to make food allergen analysis more user-friendly. When multiplex dipsticks are constructed for food allergens, they should be designed to fit the criteria of consumer-friendliness. Lateral flow immunoassays are easy-to-use, safe, affordable, portable, rapid, sensitive, and can be quantitative when linked with a dipstick reader such as a smartphone.

#### *Smartphone lateral flow immunoassay readers*

Although LFIA results can be visually detected with the naked eye, by integrating LFIA with a smartphone detector system, a quantitative result can be achieved. Owing to their simple structure, LFIAs are fairly simple to interface with smartphones, as the results can be easily detected via the phone’s camera. Smartphone dipstick readers can be categorized based on their light source; some rely on LED as the external light source whilst others utilize the internal flash in the phone.

Mudanyali et al. described a smartphone readout system termed rapid diagnostic test reader (RDS) [25]. The reader is made up of a 3D-printed 65 g mechanical attachment, which consists of: a LFIA strip holder, an inexpensive lens, three LEDs, and three AAA batteries. The device captured images of the LFIA, which were digitally processed within the related smartphone app. The linked central database received and stored the processed results in a world map through geo-time stamping. This device was validated by using commercially available malaria, tuberculosis, and HIV LFIA [25]. Another example applying LED as an external light source was described by Lee et al. for using a smartphone-based readout system integrated with a LFIA reader for the detection of aflatoxin B1 [23]. The device described a LFIA reader consisting of: a close-up lens, white LEDs, and batteries. A smartphone camera was positioned over the lens of the LFIA reader where the camera recorded images of the optical density of the LFIA test and control lines. Lee et al. further refined this LED-based format of LFIA reader and smartphone app for image capture and data acquisition for *Salmonella* detection [24]. This format of LFIA strip readers utilize LEDs as light sources, which requires external battery packs for power.

Another format of smartphone LFIA readers utilizes the smartphone’s embedded camera flash as the light source. Oncescu et al. developed a smartphone readout system for the colorimetric detection of changes of pH in sweat and saliva [106]. The device used a 3D printed phone case, which housed a slot for the indicator pH strip, a reference strip, and room to store up to six spare pH test strips. The attachment applied PDMS light diffusers to allow reproducible illumination from the camera flash. The strips were photographed and the RGB (red, green, blue) values were analyzed and converted to a hue spectrum. Hue more appropriately fits the range of color for pH strips. In another study, Oncescu et al. advanced the use of the internal flash of a phone camera for reading of LFIA for cholesterol testing [107]. This device is referred to as the smartCARD and it monitors the colorimetric change resulting from a cholesterol enzymatic interaction on a test strip. The phone flash and camera are then used to record images of the colorimetric reaction, which is then digitally processed in the related app. The attachment has a slot for the test strip and a PDMS light diffuser. The device converts recorded RGB values to hue, luminosity, and saturation values within the app and is capable of quantifying cholesterol over all physiological values [106, 107]. A further example of an embedded flash-based LFIA smartphone reader for screening thyroid stimulating hormone (TSH) was described by You et al. [108]. This device used an opto-mechanical 3D printed attachment that directed the light from the phone camera, via an optical fiber, to a collimating lens to illuminate the LFIA. The study emphasized the importance of minimizing the Mie scattering of the nitrocellulose membrane particles and maximising the Raleigh scattering of the gold nanoparticles of the test/control lines, increasing the signal in these regions. The improved signal to noise ratio allowed a very sensitive LOD to be achieved with this readout system. Although these examples have not yet been applied to allergen testing, the technology could easily be translated for allergen analysis.

Commercial companies are now finding ways to advance their traditional LFIAs by interfacing them with smartphone technology. R-Biopharm’s (Darmstadt, Germany) RIDA QUICK lateral flow assays are compatible with the RIDA SMART App, which acts as an embedded flash smartphone-based lateral flow strip reader. Currently, the mycotoxin strip test range has been converted for use with the app but it is expected that soon all RIDA QUICK assays (including the extensive allergen range) will be compatible with the app [109]. Once a sample has been tested with the LFIA, a strip cover with the color calibration required by the app to distinguish the differences in test/control line intensity, is placed over the strip. The strip and cover are placed in a cardboard enclosure; this box is to control ambient light conditions and ensure that consistent results are achievable. The app uses the smartphone camera to capture a photo of the strip. The results are automatically stored within the app database/and or can be exported to e-mail or printed via a WiFi connected/Bluetooth printer. The major benefit of the app is the ability to quantify results; however, when testing for food allergens, a semi-quantitative result would be sufficient as there are currently no set threshold levels for allergens EU legislation. Although the company also makes quantitative readers, using a smartphone is significantly more affordable and user-friendly for the general consumer. A major limitation for this set-up is that it is currently only suitable for use with the Android platform (5.1-8.0 OS) and on a limited number of smartphone models (Google NEXUS 6, NEXUS 6P, and Pixel XL) [109].

Lateral flow fits the criteria of being affordable, portable, disposable, and rapid. The popularity of using smartphones as LFIA readers has also been highlighted by commercial companies, such as Novarum and Mobile Assay, which develop bespoke smartphone apps for the reading of established LFIAs [110–113].

### ELISA

Enzyme linked immunosorbent assays (ELISA) is the most routinely used method of allergen analysis in the food industry [5, 114]. Commercially available allergen ELISAs are listed in ESM Table S3. ELISAs exist in both competitive format (suitable for low molecular weight proteins) and sandwich format, which is the prominent choice for food allergens [83]. Both formats of ELISA are based on the interaction of an enzyme labelled allergen-specific antibody with an antigen. An antibody is labelled with an enzyme, which initiates a measurable colorimetric change upon the addition of the substrate. The reaction is measured by an ELISA plate reader [115]. In sandwich ELISA, the measured response is directly proportional to the concentration of allergen in the sample. Owing to the laboratory-based nature of ELISA, which involves following a standard operating procedure and technical instructions, the requirement for scientific equipment/trained personnel and the long incubation steps, ELISA cannot be considered consumer-friendly [116]. Nevertheless, a few smartphone interfaces have been designed for use in resource limited settings.

#### *Smartphone 96-well microplate readers*

Microplate readers are one of the most used instruments in routine immunochemical analysis. However, they are relatively expensive, require maintenance, and are non-portable, making them inaccessible for in-field testing [117]. It is possible to create smartphone-based spectrophotometers using the smartphone camera [25, 117–119]. In a 2016 study, Fu et al. described the development of a smartphone-based microplate reader capable of detecting biomarkers in the absorbance range of 340–680 nm [120, 121]. This research relied upon established commercial ELISA and compared the results with microplate reader Synergy H^1^ Hybrid Multi-mode Microplate Reader (BioTek Instruments; Winooski, VT, USA) for validation. Once the assay was complete, the 96-well plate was introduced to the smartphone-based microplate reader, which was attached to the camera of the smartphone. The related app stores calibration curves that convert the transmitted light intensity to absorbance values and then to analyte concentrations [120]. The results obtained were slightly lower than with the commercial microplate reader.

Another example was described by Berg et al. from Ozcan’s group of University of California, Los Angeles (UCLA), which describes a microplate reader based on a Windows phone (Lumia 1020, Nokia) with 3D printed attachment and a data processor connected to the Cloud [117]. The colorimetric reader used a 3D printed opto-mechanical attachment with a light emitting diode (LED) to illuminate 96-well plates. The light from the LED is transmitted through 96 individual optical fibers that redirect the light to a collection lens, which then transmits the captured images of the samples to the custom-designed app for signal reading. The processing algorithm focuses on finding two centroids to use as references in the 96-well plate and pixel intensity thresholding to separate wells for independent analysis. The device was successful and was able to match the performance of a Food and Drug Administration (FDA) approved microplate reader [117].

The use of smartphones as microplate readers will make ELISA technologies more accessible; by making them portable, able to connect to WiFi, and upload results to the Cloud in real-time. This adaptation will be significantly beneficial in low resource settings such as in developing countries. As ELISA requires multiple reagent handling steps, it is necessary for the user to be able to utilize a pipette. Long incubation steps and multiple washing steps prevent the method from being consumer-friendly. Even if a smartphone app had a step by step guide showing which reagents to use at each interval, the method would still not be that consumer-friendly. The detection method on the smartphone is, however, more user-friendly in the sense that it is affordable, portable, and can connect wirelessly so it is suitable for in-field conditions.

#### Smartphone 8**-w**ell **s**trip **m**icroplate **r**eader

**Fig. 1:**
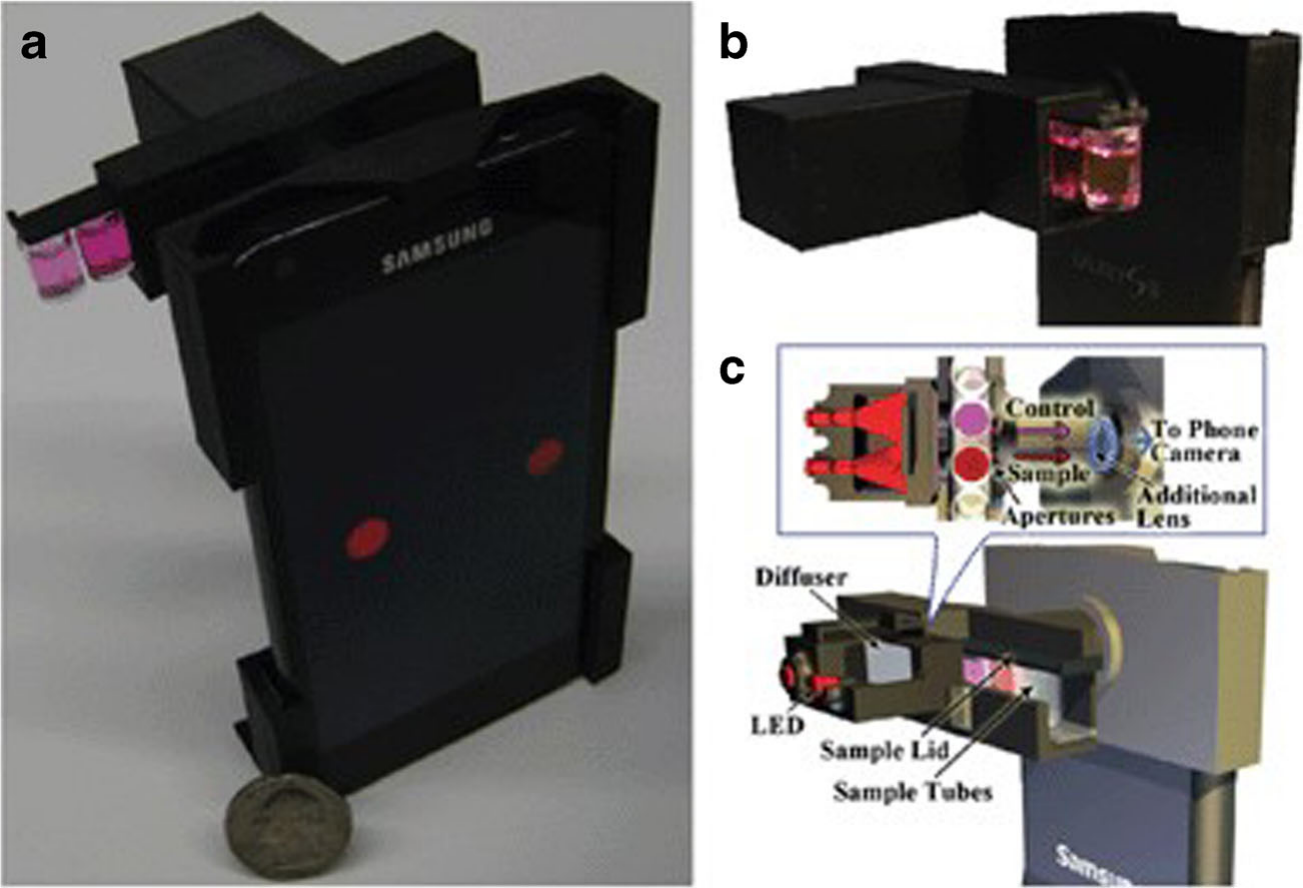
**(a)** An image of the *iTube* platform, using a Neogen Peanut ELISA 8-well strip and a smartphone-based digital reader, is displayed. **(b)** The 3D printed opto-mechanical attachment, which is connected to the rear-facing camera on the smartphone. **(c)** A schematic of the *iTube* is shown. Reproduced from [122] with permission of The Royal Society of Chemistry

In some scenarios, the user may only want to analyze a small number of samples rather than a whole 96-well plate; in these circumstances a smartphone detector that analyzes a strip of eight microwells may be more appropriate. The iTube is a novel allergen testing platform also developed by Ozcan’s laboratory at UCLA. The device is a 3D printed opto-mechanical attachment that is connected to the existing camera of a smartphone (Fig. 1) [122]. The approach is based on a 8 well strip of the commercial Neogen peanut ELISA. The platform consists of a 3D printed attachment that holds the microwells and the smartphone reader, and a related ‘iTube’ app that converts transmission images received from the camera to relative absorption values, which can be related to the concentration of allergen present within the sample [122]. The attachment weighs around 40 g and is made up of: a plano-convex lens, two LEDs, two light diffusers, and circular apertures to allow control of the field of view. Once the peanut assay has been performed, transmission intensities are recorded using the smartphone camera, and the images are digitally processed. The digital processing in the app occurs by converting the transmission images of the light through the test tubes into binary mask images. The detector is semi-quantitative, giving a positive signal for samples containing over 1 ppm peanut and negative results for lower concentrations. Another example of a single-strip 3D printed smartphone microplate reader was successfully explored by Wang et al. for the detection of herbicide 2,4 dichlorophenoxyacetic acid, which further clarifies that in some situations only a limited number of samples require analysis [119]. Like most smartphone-based analytical devices, the iTube has the ability to upload results to servers through its app. This means that a personalized allergen testing database can be constructed and users can monitor tests they have carried out on different foods, in varied locations, creating a spatio-temporal allergen map. Using anonymized ‘big data’ in this way not only assists allergic sufferers, but also helps those involved in food manufacturing, product design, and official regulators to better understand allergens from a consumer point of view.

### Flow Cytometry: Bead Suspension Array

Flow cytometry (FC) in suspension array format uses microbeads as solid phase support systems for capture antibodies to be immobilized onto. The bead–antibody complex can be identified by its fluorescent/colored profile by a flow cytometer [123]. Flow cytometry can be used for both in-vivo and in-vitro quantitative allergen analysis [124, 125]. Garber et al. and Cho et al. have shown that by using magnetic bead sets it is possible to detect 14 food allergens (and gluten) in 12 different samples, within 6 h, with a similar LOD to existing ELISA methods (<5 ng/mL) [97, 126]. However, their methods required two extraction procedures, so although the assay could be multiplexed, the extraction could not. Otto et al. combined a competitive format ELISA with flow cytometry (BD Accuri C6 apparatus, Becton-Dickinson, Vianen, The Netherlands) to develop an assay capable of detecting five different allergens in a cookie matrix [127]. The assay could detect in the range of 2–10 ppm all the allergens in the test. Cho et al. further described the usefulness of FC for cross-reactivity profiling between 23 legumes and 12 tree nuts [128].

#### *Miniaturized flow cytometers*

Despite their success, flow cytometers (FCs) are not portable, are relatively expensive, require trained personnel, and are therefore not suitable for in-field analysis. In response to this, FC was miniaturized. Miniaturization of FC involves focusing the flow of the particles to be analyzed within a microfluidic channel, reducing the size of both the microfluidics and the optics, and integrating them with a signal readout device [129].

**Fig. 2:**
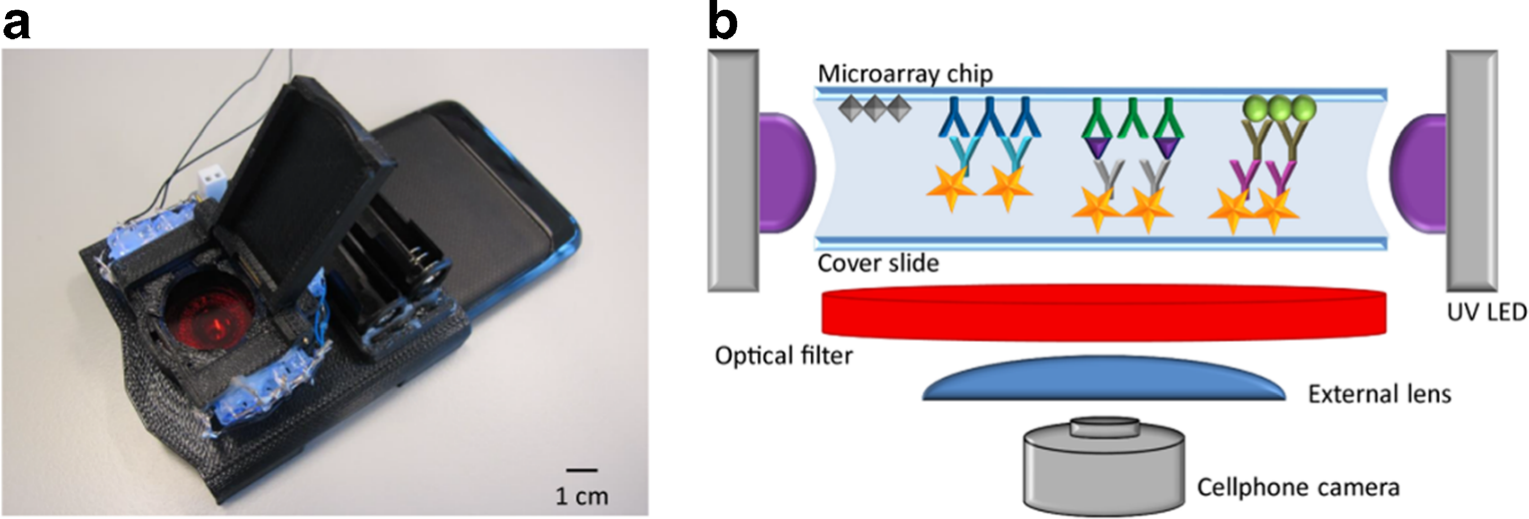
**(a)** Photo of 3D printed optical attachment on the smartphone used for testing. **(b)** Schematic representation of the smartphone biomarker detection platform. Reproduced with permission from authors [134]

The portability of miniaturized flow cytometry (MFC) makes it an attractive technique for in-field routine analysis. Connecting MFC to a smartphone readout system further strengthens its portability. Ozcan’s UCLA group have worked since 2008 to develop on-chip cytometers that are capable of interfacing with smartphones as the detector [130]. Zhu et al. have further substantiated the ability to combine MFC and optical microscopy with a smartphone interface [131]. The study integrated a microfluidic chip with a syringe pump that controlled the transport of sample to the imaging field, where a photo was captured by a smartphone camera. This example uses an opto-mechanical attachment, featuring: simple lenses, plastic color filters, LEDs, and batteries. Further development on this study yielded a smartphone-based MFC interfaced with an optical-microscope for the counting of fluorescently labelled blood cells [132]. Despite these examples being for the healthcare sector, they provide an excellent basis for future design of smartphone based cytometers for application to food allergen analysis. Similarly, MFC has been used in contaminant and residue monitoring in milk samples [133]. An assay was designed to detect growth promotor bovine somatotropin (rbST). Biomarker-specific antibodies (anti-rbST) were coupled with quantum dots (QD), which were immobilized on paramagnetic microspheres. The device relied on an optical-mechanical attachment consisting of a phone holder (for alignment of optics), a sample tray to hold the cover slides, 12 UV excitation LEDs, white LEDs, an optical filter, a de-magnifying lens, and a lid to prevent introduction of ambient light [133]. The smartphone camera was used to record images of the fluorescence emitted from the QD. This assay still takes a substantial amount of time to carry out owing to incubation steps so it cannot be classified as a rapid assay. An even more sophisticated multiplex smartphone approach based on the original rbST microsphere assay was presented for biomarkers in milk (Fig. 2) [134]. Although this technology has currently only been applied to food diagnostics, focusing this approach could allow it to be applied more specifically to food allergens.

### Multiplex surface plasmon r**esonance**-based food allergen biosensor

This review has averted biosensors, due to the in-depth review on using biosensors for food allergen analysis published in 2016 and another 2016 review focusing specifically on smartphone-based biosensors [9, 28]. Only brief attention will be paid here to biosensors. Surface plasmon resonance (SPR) monitors changes in the refractive index based on the dielectric properties of a thin layer of sample containing solution, near the gold metal surface of the sensor region. The energy transfer from polarized light to surface plasmon results in characteristic reflected light patterns that can be monitored label free, in real-time through a sensorgram (the angle at which the dip is observed versus time) [135]. Analyte-specific antibodies are immobilized onto the metal layer of the sensor chip, mounted onto a glass prism with an integrated flow cell that is then placed in the instrument. When polarized light shines through the prism, the light is reflected by the metal layer, resulting in an angle of incidence capable of inducing surface plasmon resonance and causing a dip in the reflected light intensity [136]. The refractive index near the metal surface will change as proteins are adsorbed onto the metal surface and the amount of adsorbed protein can then be determined. Unfortunately, current ‘portable’ SPRs still require a laptop or small computer to operate [137].

Imaging SPR (iSPR) has the benefit of being able to simultaneously detect multiple analytes in a single sample. Raz et al. described an iSPR linked with an allergen-antibody array for the detection of 12 food allergens within 12 min [138]. The rapid, multi-analyte method is quantitative and detects food allergens at 2 mg/kg. The procedure allowed for total allergen profiling within food, providing a unique fingerprint for which allergens each commercially available food contained. The optical devices laboratory of Linköping University (Sweden) described a smartphone-based angle resolved localized SPR device [139]. The device used the phone screen as the light source, the phone camera to record images, and a disposable optical coupler made of PDMS/epoxy, which matched the refractive index of glass [139]. The polymer surface contained glass coated with a layer of gold, as the thin metal surface, with which simple or more complex microfluidic systems are compatible. The app allowed a red rectangle on the phone screen to frame the region of interest (ROI) to be photographed; which ensured that the images were all captured under the same conditions, in the right ROI, minimizing test-to-test variation. The camera shutter and exposure were set using a simple app developed for iOS 5. When the light was reflected from the gold chip surface, the SPR signal was transported to the front camera of the phone, where it was conditioned by deflection via a PDMS prism. The method was validated using a commercial β-microglobulin assay but should be compatible with numerous other targets.

Guner et al. described interfacing a smartphone with disposable Blu-ray discs as SPR chips and a 3D printed iSPR attachment [26]. Detection limits were reported as comparable with commercial instruments. The SPR attachment recorded measurements from over 20,000 individual pixels based on an intensity interrogation mechanism. An additional study applying fiber-optic SPR (FO-SPR) using a smartphone platform has achieved results consistent with commercial SPR instruments [140]. Although the FO-SPR interfaced with the smartphone is portable and allows precise detection and sophisticated optical calibration, owing to the need to compensate for alignment issues in the app, the platform cannot be classified as consumer-friendly.

### Section summary

The food allergen detection methods that have been discussed so far do not fully satisfy the criteria for consumer friendliness and are therefore not currently suitable for citizen science. In order to be consumer-friendly, the technique should be easy to carry out, requiring minimal training. Of the methods discussed so far, dipsticks are generally considered to be user-friendly with many people being accustomed to using home pregnancy tests, which are historically the first example of LFIA [100]. The majority of the population would be capable of carrying out a strip test with minimal instruction, and when linking the test to a smartphone reader, would be able to interpret the results. However, smartphone dipstick readers have not yet been developed for food allergen detection and although the general consumer could carry out the LFIA easily, they would not have a quantitative strip reader so the results would only be qualitative. But for food allergen analysis, it is not fundamental to have a quantitative result as long as the result is semi-quantitative within a small range, as there are currently no set threshold levels for food allergens (excluding gluten). If consumers wanted to use their screening results in court, for example to sue a company for the presence of an undeclared allergen, it would first be necessary to use orthogonal approaches to confirm the result with instrumental analysis such as mass spectrometry anyway [141].

Whilst LFIA are simple to carry out, methods such as ELISA, FC, and SPR all require training to perform. The methods require understanding of laboratory practice and experience in data interpretation to achieve meaningful results. Even when linking with a smartphone readout system, ELISA still requires laboratory skills, such as using precision pipettes, to carry out. Performing an ELISA is time-consuming owing to the incubation steps and need for external equipment. As the assay uses open test tubes, it is possible that there could be spillage of chemicals, which would mean the user carrying out the test would require PPE, which further limits its potential as a user-friendly device. ELISA has the disadvantage of currently being non-reusable, non-recyclable, and produces chemical waste. Flow cytometry is a multiplex laboratory-based method, meaning that it is not portable. It requires scientific skill and good laboratory practice to stay safe, and uses expensive instrumentation. The advancement of MFC with a smartphone-based readout makes FC more user-friendly by providing an inexpensive platform, which can be easily operated and reused, decreasing the cost of the assay. An additional benefit of MFC is that it is portable and therefore can be used in the field. Of the discussed methods, smartphone SPR may be the most promising for citizen science as it has the benefit of having limited sample preparation steps owing to its label-free nature, and results in real time and the ability to reuse the sensor chip. Interfacing with a smartphone also makes SPR portable and suitable for in-field use.

All of the methods, except for LFIA and SPR, require trained personnel, take a prolonged period of time to carry out, have complex data acquisition, and need to be completed under laboratory conditions. This means that the general population would not be able to efficiently carry out these tests and so they cannot be classified as user friendly.

## Consumer-friendly by design

Whilst the previous section discussed scientific methods for food allergen analysis, this section will focus on methods that have specifically been designed with the intention of being consumer-friendly. The devices are compared in Table 2 below. Consumer-friendly devices are needed as allergic individuals require devices that can be easily operated whilst at home or in a restaurant. Consumer-friendly detectors will allow the road to be paved for citizen science, as the general population of allergic sufferers will be able to perform their own food analysis.

**Table 2:** Consumer-friendly by design: comparison of devices

	Smartphone Readout	Consumer-friendly by Design		
Criteria	RIDA Smart App	NIMA	Allergy Amulet	iEAT
Safe	**Y**	**Y**	**Y**	**N**
Portable	Y	Y	Y	Y
Quantitative	Y	Y	Y	Y
Total speed (min)	<10	<3	Not stated	<10
LOD (mg/kg)	Low mg/kg range (dependent on assay)	2	1–2	Gliadin: 0.075 mg/kg Ara h1: 0.007 mg/kg Cor a1: 0.089 mg/kg Caesin: 0.170 mg/kg Ovalbumin: 0.003 mg/kg
Multiplex?	N	N	Not stated	Y (x5)
Extraction	Pre-analysis with extraction buffer and shaking	Internally in capsule	Stated as ‘Not necessary’	2 min incubation with TECP/sarkosyl at 60^°c^
Sample prep	Homogenise sample	Internally in capsule	Stated as ‘Not necessary’	Food extract mixed with Ab solution; transferred to PBS; incubated with HRP-conj Ab; mixed with TMB; loaded onto electrode
Mechanism	LFIA strip reader with smartphone display	LFIA strip reader with sensor display	MIP strip reader with sensor display	Magneto-electrochemical sensing with an electronic keychain reader
Connectivity	WiFi, Bluetooth	WiFi, Bluetooth (through App)	WiFi, Bluetooth (through App)	Bluetooth & Smartphone app
Cost	€12.75 per strip test (box of 20) & €150 and then €80 per year for app	$279 + $5 for each use	Not Stated	<$40 for device & <$4 per antigen
Validation	N	Yes, against R-Biopharm	N	Potentiostat SP-200 Bio-Logic using potassium ferrocyanide standard solution

### Portable gluten sensor

NIMA (San Francisco, CA, USA) is a commercial portable gluten detector based on a immunochromatographic dipstick and a sensor. The device provides a testing platform for individuals with celiac disease/gluten intolerance to be able to perform their own gluten analysis.

The device is portable, sensitive, and rapid, taking only 2 to 3 min for a result in the consumer-friendly form of a LED smiley face (gluten-free) or a wheat grain (containing gluten) [142]. It has fully integrated sample handling inside single-use test capsules, which makes it attractive for the general consumer, especially when considering its use in a setting such as a restaurant. The test is based on gluten antibodies (13-F6 and 14-G11) against the toxic 33-fragment of the protein, which have been immobilized as the test line of the strip test [142]. This is the fragment widely considered to be responsible for the autoimmune effects of gluten, so its detection is crucial [143]. The majority of celiac/gluten-intolerant individuals can tolerate gluten levels up to 20 ppm, and the assay detects below this level [144, 145]. However, it should be considered that if analyzing whole grains for gluten, contamination is localized to particular ‘hot spots’ rather than being ubiquitous to the whole sample, which could result in false-positives/negatives with the sensor, so it is necessary to first homogenize the sample before testing [146].

To operate the device, the user puts some chopped food into the one-use capsule. Once the food is inside the capsule, the user turns the head of the capsule operating the grinding mechanism and homogenizing the food. The final twist of the lid introduces the food homogenate to the pre-contained extraction buffer and an internal rotating motor acts as a mini-centrifuge to mix the food and buffer, solubilizing and extracting any gluten from the food [142]. After a few min, the electronic sensor will determine whether there is gluten present in the sample. An algorithm then converts this information to a smiley face icon for gluten-free or a wheat icon for products containing gluten. The sensor costs $279 and includes three one-use buffer containing capsules, a charger, and a carrier pouch [147]. Each single-use capsule can only test the food portion that you put into the capsule. To test multiple components of a meal at a restaurant, a user would need multiple test capsules, increasing the overall cost of the meal.

This set-up can be considered as user-friendly in the sense that the assay is easy to use, the results are easily interpretable, and it is safe, rapid, sensitive, and portable. NIMA has a related app that allows consumers to create a map of local restaurants or compilation of products that are truly gluten-free, which can help lessen the economic and restrictive burden of an avoidance diet. NIMA has a large social media presence, utilizing the hashtag *#nimatested* to denote restaurants and foods that have been tested using the device. The use of social media allows users of the device to communicate and opens a discussion between gluten-intolerant individuals. In addition, the product website has a wealth of information on how to operate the device, what can and cannot be tested, limits of detection, and a customer support service. A major disadvantage is the overall cost of the device, which will prevent it from becoming the first choice for every gluten-intolerant consumer; although the sensor is reusable, the one use capsules are not and cost $5 each. An additional disadvantage is that it cannot be multiplexed and its designers are making a separate sensor and assay for major peanut allergens, which further increases the cost to the consumer, particularly if they suffer from co-allergies. As a result of lack of published validation studies it is plausible that false negatives could prove dangerous to individuals with celiac/gluten intolerance and false positives from the sensor could adversely affect the food industry [79]. Lack of evidence-based literature surrounding the product makes it difficult to assess its reliability.

### Molecularly imprinted polymer allergen sensor

The Allergy Amulet (AA; Boston, MA, USA) is a rapid, portable food allergen and ingredient detection device that is currently being developed for commercial release in the winter of 2018 [148]. This device has been included in this review as a state-of-the-art consumer targeted allergy detection and management device. The device is initially being designed to target peanut protein in the concentrations of 1–2 ppm. The device uses molecularly imprinted polymers (MIP) which are synthetic receptors that can be designed to recognize a specific target allergen [149]. If the allergen is present, the selective cavities in the MIP capture the allergen through a ‘lock and key’ mechanism, and a signal on the device then alerts the user to the allergen presence [148]. The device works in theory by inserting a test strip probe directly into the food or liquid to be assessed. The website states that no sample preparation is required; however, this is difficult to believe when considering inserting a probe into samples such as peanuts or cookies. Following exposure to the sample, the probe is then inserted into its MIP containing covering sheath, and then the sheath is inserted into the amulet reader, which resembles a USB stick. If the target allergen is present, an LED in the amulet case will light up, promptly alerting the user to the allergen presence within a matter of seconds. The results are also sent via a smartphone interface to the AA app, which allows users to compare test results, creating a personalized allergen database. This helps users to connect with other food allergic individuals and compare results based on what they have eaten. It is truly portable and can be worn as a necklace or keychain. This device is consumer-friendly in the sense that it is portable, (claims to) require minimal sample preparation/extraction, and is quick, sensitive, and selective. However, as there is not sufficient evidence-based information available about the cost, reusability, and the validation/benchmarking of the device at this stage, it is impossible to state how suitable it is for citizen science.

### Portable electrochemical multiplex allergen sensor

**Fig. 3:**
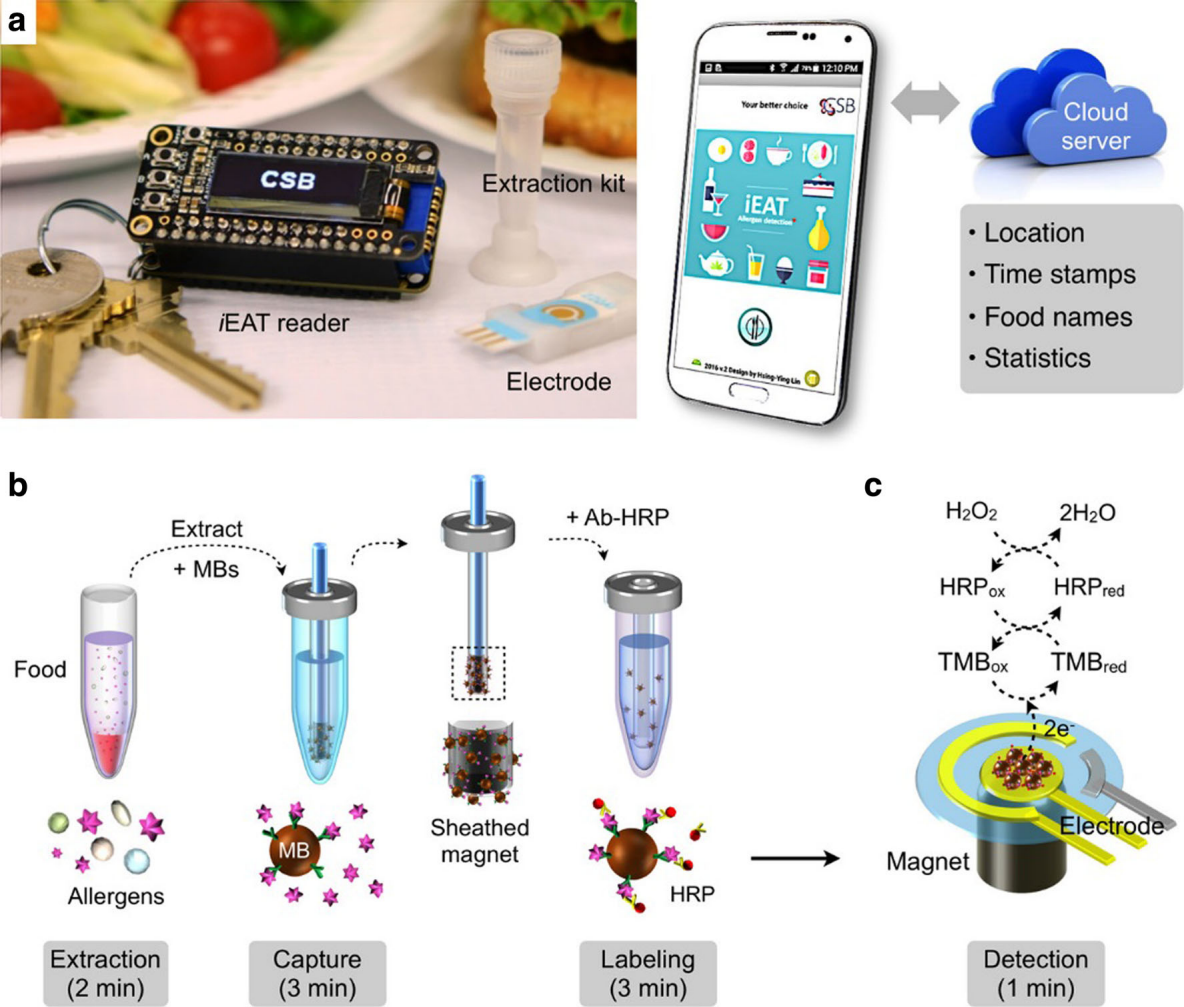
The iEAT platform. **(a)** The keychain-sized detector, the multi-channel electrode chip, and the disposable extraction kit, which is linked with a smartphone app as the readout system. **(b)** Antigen extraction; antigens are captured on magnetic beads (MB) and labelled with allergen-specific antibodies labelled with oxidizing agent HRP (horseradish peroxidase). The disposable kit contains a sheathed magnetic bar, which collects and relocates MBs. **(c)** Signal detection is achieved by mixing HRP-labelled MBs with substrate (TMB, 3,3’,5,5’-tetramethylbenzidine) and moved to the electrode. The HRP catalyses the oxidation of TMB. When TMB is oxidized (ox) or reduced (red) on/near the electrode, measurable electrical currents are given off. Reproduced with permission from [150]. Copyright 2017 American Chemical Society

The integrated exogenous antigen testing (iEAT) is a state-of-the-art, electrochemical, magnetic bead-based food allergen detection sensor. It works by conjugating the desired allergen antibody onto a magnetic bead [150]. The bead suspension containing the immobilized antibodies is then incubated with the extracted food for around 3 min before re-suspending with horseradish peroxidase (HRP)-conjugated isotype IgG antibodies, as a label. The HRP-bead complex can then be mixed with substrate (TMB) and added to the electrode. The entire assay takes less than 10 min, including extraction time. The iEAT allows singleplex or multiplex analysis when using the multichannel electrode, which can detect up to with different allergens (Fig. 3). The current device tests for major allergenic proteins (see Table 1; s.2.1) in peanut, hazelnut, wheat, egg-whites, and milk.

The assay includes a disposable extraction kit, which allows immuno-magnetic enrichment of the allergen antigens concentrating food antigens from food. The kit contains a disposable extraction device and the extraction buffers and wash solutions that are needed in pre-measured volumes. The lid of the extraction vial has a magnetic sheathed bar attached to allow for capture of allergen-magnetic beads. This bar allows easy transfer of the antibody–bead complex to the washing/labelling stages and then for loading onto the magnetic electrode, making sample handling easier for the consumer. The reader centers around a microcontroller unit linked with digital-to-analog converters and a potentiostat, which controls the potential difference between the reference and working electrodes. The sensor was benchmarked against the commercial potentiostat SP-200 Bio-Logic (Seyssinet-Pariset, France) and the two systems were reported to be excellently matched [150]. The reader is operated via a Bluetooth connection to a related Android app. The app also takes sample photos and records data such as time-stamp, analyte concentrations, and GPS information. The research suggests that a future development of the test will be to use the pressure-sensitive screen of the phone as a weighing scale. By reducing the need for extra equipment/instrumentation the consumer-friendliness of the device will be even further improved.

The low cost of the assay, the speed, and the ability to be multiplexed, orientate the assay to consumer-friendliness. The use of a magnetic bar for the transfer of the target to each step of the assay eliminates the need for the use of precision pipettes, making it more accessible to non-scientists. However, in addition to multiple sample handling steps, the assay uses harmful mutagenic chemicals such as TMB, and so would need to be carried out under careful lab conditions with PPE. The electronic key chain sensor is reusable and the extraction device is disposable. However, the assay would produce toxic waste, preventing it from being environmentally friendly and limiting its consumer-friendliness as the general user will not be accustomed to disposing of chemical waste.

## Conclusions and future perspectives

This review has targeted the recent advances toward citizen science through immuno-based food allergen analysis, with a particular focus on novel smartphone-based detection strategies. Traditional immunochemical detection methods for food allergens have been assessed for consumer-friendliness. Applying smartphone-based technologies to traditional lab-based immunochemical methods has been explored. This review has underlined the necessity for more user-focused assays that can be based on smartphones for simple food allergen analysis. By providing an easy to use, safe, affordable, portable, smartphone-based, rapid, sensitive, and multiplexed assays, citizen science can be achieved.

The popularity of using smartphone-based analytical devices has greatly improved in recent years, as can be ascertained by the increasing number of publications on the subject. However, there are still a number of developments that can be made to improve the capabilities of smartphones as detectors. One area that needs to be addressed in every smartphone-based assay is the control of ambient light conditions. Many authors have attempted to control light by using an attachment, such as a box which controls the field of light, or a lid on a 3D printed attachment which means that photos can be captured in consistent conditions. Alternatively, it has been suggested that a more appropriate way to control differences in lighting conditions would be to include a normalization algorithm in the app to allow optimum image capture through controlling the lighting bias [12]. Currently, most assays/apps are based on a singular platform, but for a detector to be truly consumer-friendly it should be compatible with all the major smartphone platforms (iOS, Android, and Windows) so that the user does not need to purchase a specific model. Future developments should concentrate on making a multi-platform system. It must be considered when transferring from one model or platform to another that smartphone models have variance in the number of megapixels, different positions of their front/rear facing cameras, and altered position of their flash.

Future devices should aim for embedded storage of pre-contained dry reagents so that minimal user interference is required. Future applications should focus on designing a sampling interface that would allow the sample collection and detection to be carried out in one device. Such an integrated device could limit sample preparation steps as these could be carried out within the attachment, greatly improving its consumer-friendliness.

This review has shown that despite the current lack of truly consumer-orientated devices, the allergen diagnostics industry is taking the first steps to become more user-friendly. Devices such as NIMA, AA, iEAT, and the RIDA smartphone range are designed with the consumer in mind and exemplify the change in attitude in industry to move towards citizen science. Food allergies are personal, and by engaging the consumers with their own diagnostic analysis, food allergen analysis will be improved, as more people will take responsibility for their own food safety and big data can be collected. Currently the burden for food allergies lies heavily on food manufacturers and labelling legislations, but by developing devices that can detect multiple allergens in a sample, consumers can take analysis into their own hands. It is desirable for standardized reference materials for both raw and processed allergens to be developed and utilized at assay development stages so that consumer-friendly devices can be properly benchmarked and validated. Having well validated consumer-friendly assays paves the way to the future of citizen science.

## Electronic supplementary material

ESM 1 (PDF 232 kb)

## Abbreviations

AA: Allergy Amulet
ABA: Allergen Bureau of Australia and New Zealand
ALARA: As low as reasonably achievable
CCD: Charge coupled device
CIP: Clean in place
EC: European Commission
ELISA: Enzyme linked immunosorbent assay
FARRP: Food Allergy Research and Resource Program
FC: Flow cytometry
FDA: Food and Drug Administration
FO-SPR: Fibre optic surface plasmon resonance
GPS: Global positioning system
HRP: Horseradish peroxidase
Ig: Immunoglobulin
LED: Light emitting diode
LFIA: Lateral flow immunoassay
LOAEL: Lowest observable adverse effect limit
MFC: Miniaturized flow cytometry
MIP: Molecularly imprinted polymers
NOAEL: No observable effect limit
PAL: Precautionary allergen labelling
PDMS: Polydimethylsiloxane
PoC: Point-of-care
PPE: Personal protective equipment
ppm: Parts per million
QD: Quantum dots
ROI: Region of interest
(i)SPR: (imaging) Surface plasmon resonance
USB: Universal serial bus
UV-VIS: Ultraviolet visible spectrophotometry
VITAL: Voluntary incidental trace allergen labelling
