# Supplementary material for: Consumer-friendly food allergen detection: moving towards smartphone-based immunoassays
Source: Anal Bioanal Chem. 2018 Mar 26;410(22):5353–71. doi: 10.1007/s00216-018-0989-7 (PMC6096701; doi:10.1007/s00216-018-0989-7)
Supplement: Supplementary file 1 — (PDF 232 kb) [file 216_2018_989_MOESM1_ESM.pdf]

Analytical and Bioanalytical Chemistry

Electronic Supplementary Material

Consumer-friendly food allergen detection: moving towards smartphone based immunoassays

Georgina M. S. Ross, Monique G. E. G. Bremer, Michel W. F. Nielen

Contents

Table S1 Food allergens requiring labelling in different countries 3
Table S2 Proof-of-concept Multiplex Allergen Assays 4
Table S3 Commercially Available Food Allergen Assays 7
Table S4 Conventionally lab-based allergen detection methods and their smartphone-based counterparts 34
References 36

Table S1 Food allergens requiring labelling in different countries

	EU	Canada	USA	Australia/New Zealand	China	Hong Kong	Japan	Korea	Taiwan	Argentina	Thailand	Bolivia	Brazil	Chile	Colombia	Costa Rica	Cuba	Mexico	Nicaragua	South Africa	Venezuela
Milk	X	X	X	X	X	X	X	X	X	X	X	X	X	X	X	X	X	X	X	X	X
Eggs	X	X	X	X	X	X	X	X	X	X	X	X	X	X	X	X	X	-	X	X	X
Cereal/ Wheat	X	X	X	X	X	X	X	-	-	-	X	-	-	X	X	X	X	X	X	X	X
Peanut	X	X	X	X	X	X	X	X	X	X	X	X	X	X	X	X	X	X	X	X	X
Tree Nuts	X	X	X	X	X	X	-	-	X	X	X	X	X	X	X	X	X	X	X	X	X
Fish	X	X	X	X	-	X	-	-	X	X	X	X	X	X	X	X	X	X	X	X	X
Crustacean	X	X	X	X	X	X	X	X	X	X	X	X	X	X	X	X	X	X	X	X	X
Soy	X	X	X	X	X	X	-	-	X	X	X	X	X	X	X	X	X	X	X	X	X
Celery	X	-	-	-	-	-	-	-	-	-	-	-	-	-	-	-	-	-	-	-	-
Mustard	X	X	-	-	-	-	-	-	-	-	-	-	-	-	-	-	-	-	-	-	-
Lupin	X	-	-	-	-	-	-	-	-	-	-	-	-	-	-	-	-	-	-	-	-
Sesame	X	X	-	X	-	-	-	-	-	-	-	-	-	-	-	-	-	-	-	-	-
Sulphites	X	X	-	-	-	-	-	-	X	X	X	X	X	X	X	X	-	X	X	-	X
Other	-	-	-	-	X	-	X	X	X	X	-	X	-	-	-	-	-	-	-	-	-

Table S2 Proof-of-concept Multiplex Allergen Assays

Assay Format	Allergens Detected	Label Used	Extraction Buffer	LOD	Ref
iSPR	Peanut, hazelnut, soy, casein, egg, pine nut, macadamia nut, brazil nut, cashew, pistachio & pecan.	Label free	RIDASCREEN allergen extraction buffer (R-Biopharm AG)	0.4-4.6 mg/kg	[1]
Compact Disc Immunoassay	Gliadin, casein, B-lactoglobulin & ovalbumin	Gold nanoparticle amplified by silver enhancement method	40% ethanol in PBST (pH 7.5)	0.04-0.16 mg L^{-1}	[2]
Reverse dot blot immunoassay	Peanut, hazelnut & brazil nut	Biotin	PBS with 0.1% (v/v) tween 20 & 4.0% (w/v) skimmed milk powder	0.01-0.02 mg/kg	[3]
Microfluidics ELISA-based optical sensor	Ara h1 & Gluten	Horseradish peroxidase	Extraction buffers provided in ELISA kits (Indoor Biotechnologies for Ara h1 and Crystal Chem for Gluten)	4.77-15.2 ng/mL	[4]
Sandwich assay format optical biosensor	Milk, egg, hazelnut, peanut, shellfish & sesame	Label Free	20mM TRIS pH 8.7, 150mM NaCl	1-12.5 µg/kg	[5]
Flow Cytometry	Casein, soy & gluten	Coloured super-paramagnetic carboxylated microbeads	2-ME in guadine HCl in PBS	Not Specified	[6]
Flow Cytometry	Crustacean, peanut, egg, milk & mustard	Coloured super-paramagnetic carboxylated microbeads	PBS (pH 7.5) NaCL (50mM), Na_2HPO_4 (40mM), KH_2PO_4 (5mM) & NaN_3 (1mM)	2-10 mg/kg	[7]
Flow Cytometry	Crustacean, egg, gluten, milk, peanut, soy, almond, brazil nut, cashew, coconut, hazelnut, macadamia, pine nut, pistachio & walnut	Coloured super-paramagnetic carboxylated microbeads	PBS-T (nondenatured) and 0.5% SDS/2% B-mercaptoethanol (denatured)	5 ng/mL	[8]
Flow Cytometry	Soy, pea & soluble wheat proteins	Coloured super-paramagnetic	20mL PBS (pH 7.4)	0.5-0.6 µg/mL	[9]

		carboxylated microbeads (using red and orange fluorophores)			
Optical thin film biochips	Mustard, lupine, walnut, hazelnut, celery, almond, oat & sesame	Label Free	CTAB extraction buffer (2% CTAB, 1.4 mol/l NaCl, 01.mol/l Tris/HCl, 20 mmol/l Na_2EDTA pH 8.0)	0.001%	[10]
DNA Microarray on a DVD	Hazelnut, peanut & soybean	Label Free	CTAB extraction with 10uL RNAse and 10uL proteinase K & column purification	1 µg/g (0.0001%)	[11]
Decaplex PCR with capillary electrophoresis	Hazelnut, peanut, pistachio, oat, sesame, cashew, barley, wheat, soybean & pecan	Label Free	Plant genomic DNA isolation kit; and 1.0% agarose gel for electrophoresis	0.005%	[12]
Tetraplex real-time PCR	Soy bean, celery, white mustard & brown mustard	Fluorophore	CTAB extraction buffer 20 g/L, 1.4 mol/L sodium chloride, 0.1 mol/L TRIS, 0.02 mol/L Na_2EDTA (99+%) pH 8.0 and 60uL proteinase K	2.6-36.8 mg/kg	[13]
Real time PCR	Mustard, celery, wheat & rye	Fluorophore	CTAB extraction buffer & incubation with proteinase K lysates centrifuged & purified with chloroform & isoamyl alcohol	5-40 mg/kg	[14]
Real time PCR	Sesame, almond, lupine & brazil nut	Fluorophore	CTAB extraction with precipitation step	10 mg/kg	[15]
Real time PCR	Citrus Fruits	Fluorophore	Trizol to extract RNA. DNA digested with DNase1. Total RNA reverse transcribed with random hexamers using Prime-Script first strand cDNA synthesis kit	20 fg DNA per PCR tube	[16]
Real time PCR	Almonds, hazelnuts, cashews, peanuts & sesame seeds	Fluorophore; SYBR®GreenER™	CTAB extraction	0.5-5 pg of DNA	[17]

One step PCR	Brazil nut & pecan	Not stated	DNeasy plant mini kit column	0.1%	[18]
Multiplex ligation dependent probe amplification (MLPA)	Sunflower seed, poppy seed, flaxseed, sesame & soy	Fluorophore	Wizard DNA clean-up system kit	10 mg/kg	[19]
Multiplex ligation dependent probe amplification (MLPA)	Sesame, soy, hazelnut, peanut, lupine, gluten, mustard & celery	Fluorophore	DNeasy plant mini kit column	0.001%	[20]
Multiplex ligation dependent probe amplification (MLPA)	Peanut, cashew, pecan, pistachio, hazelnut, sesame seeds, macadamia nut, almond, walnut & brazil nut	Fluorophore	CTAB extraction: 2% CTAB, 1.4 M NaCl, 20mm EDTA, 100mM TRIS-OH/HCl and 30µl proteinase K at 65°c overnight	5 mg/kg^{-1}	[21]
Multiplex ligation dependent probe amplification (MLPA)	Scallop, fish, gastropod, crustacean, cephalopod & bivalve	Fluorophore	CTAB: 2% CTAB, 1.4 M NaCl, 0.1 M TRIS & 20mm EDTA & proteinase K solution at 65 ^{o}c overnight	20-100 mg/kg^{-1}	[22]
Giant magneto-resistive sensor array	Peanut (Ara h1 & Ara h2) & gliadin	Streptavidin conjugated to magnetic microbeads and biotinylated detection antibody	N/A	0.2-7 ng/mL	[23]

Table S3 Commercially Available Food Allergen Assays

Allergenic Food	Product	Target	Assay Format	LOD/LOQ	Time (min)	Company
Cereals with Gluten	RIDASCREEN Gluten	Gliadin/gluten	Sandwich ELISA	0.5mg/kg gliadin; 1.0mg/kg gluten	90	R-Biopharm AG
	RIDASCREEN FAST Gluten	Gliadin/gluten	Sandwich ELISA	0.5 mg/kg gliadin; 1.0 mg/kg gluten	30	R-Biopharm AG
	RIDASCREEN Gluten Competitive	Gliadin/gluten	Competitive ELISA	2.3 mg/kg gliadin; 4.6 mg/kg gluten	40	R-Biopharm AG
	RIDAQUICK Gluten	Gliadin/gluten	Lateral Flow Strip	1.3 µg/100cm2 gliadin on surfaces; 2.2 mg/kg gliadin; 3.1 mg/kg gluten	5	R-Biopharm AG
	Gliadin/Gluten ELISA	Gliadin/gluten/wheat/barley	Sandwich ELISA	0.3 mg/kg	60	Immunolab
	Gliadin ESGLISS-48	Gliadin	Sandwich ELISA	2.0-20 mg/kg	Not Stated	ELISA Systems
	VERATOX for Gliadin	Prolamins (wheat gliadin, rye secalin & barley hordein)	Sandwich ELISA	5 mg/kg	30	Neogen

	VERATOX for Gliadin R5	Prolamins (wheat gliadin, rye secalin & barley hordein) in finished food and clean in rinse	Sandwich ELISA	2.5 mg/kg	30	Neogen
	REVEAL 3-D for Gliadin	Gliadin	Lateral Flow Strip (with overflow line)	5 mg/kg; (10ppm gluten); 2 µg/100cm2 gliadin	5	Neogen
	REVEAL 3-D for Gluten	Gluten	Lateral Flow Strip (with overflow line)	5-10 mg/kg; 80 µg/100cm2 swab	5	Neogen
	ALERT for Gliadin	Gliadin/gluten	Sandwich ELISA	10mg/kg gliadin; 20mg/kg gluten	30	Neogen
	ALERT for Gliadin R5	Gliadin/gluten in processed foods/CIP/environmental surfaces	Sandwich ELISA	10 mg/kg gliadin; 20 mg/kg gluten	30	Neogen
	PROTEON for Gluten	Gliadin/gluten	Sandwich ELISA	LOD: 2.5 mg/kg LOQ: 8.9 mg/kg	90	Zeulab
	PROTEON EXPRESS for	Gliadin/gluten	Lateral Flow Strip	3 mg/kg	10	Zeulab

	Gluten					
	EZ™ Gluten	Prolamins & Glutelins of wheat, rye, barley & related grains	Lateral Flow Strip	10 mg/kg	17	ELISA Technologies Inc.
	ALLER-TEK™ Gluten ELISA	Prolamins & Glutelins of wheat, rye, barley & related grains	Sandwich ELISA	5 mg/kg	160	ELISA Technologies Inc.
	Gluten (Gliadin) ELISA Kit	Gluten, Wheat	Sandwich ELISA	0.26 mg/kg gluten; 0.31 mg/kg wheat	Not Stated	Crystal Chem
	Gluten (Gliadin) Lateral Flow Kit	Gluten, Wheat	Lateral Flow Strip	5 mg/kg wheat; 4 mg/kg gluten	15	Crystal Chem
	Agrastrip® Gluten	Gluten	Lateral Flow Strip	4 mg/kg	11	Romer Labs
	AgraQuant® Gluten	Gluten	Sandwich ELISA	2 mg/kg	60	Romer Labs
	Agrastrip® Gluten G12	Gluten	Lateral Flow Strip	3 mg/kg	11	Romer Labs
	AgraQuant® Gluten G12	Gluten	Sandwich ELISA	0.6 mg/kg	60	Romer Labs

	Monotrace Gluten ELISA	Gluten/Gliadin	Sandwich ELISA	LOD: Gluten; 0.3mg/kg; Gliadin: 0.15 mg/kg LOQ: Gluten: 2 mg/kg; Gliadin: 1 mg/kg	40	BioFront Technologies
	GlutenTox Home	Gluten	Lateral Flow Strip	5 mg/kg	<20	Biomedal Diagnostics
	GlutenTox Pro	Gluten (wheat, barley, rye and OAT)	Lateral Flow Strip	5 mg/kg	<20	Biomedal Diagnostics
	GlutenTox Sticks Plus	Gluten (G12 antibody)	Lateral Flow Strip	3 mg/kg	<20	Biomedal Diagnostics
	GlutenTox ELISA	Gluten	Sandwich ELISA	0.6 mg/kg	150	Biomedal Diagnostics
	GlutenTox ELISA	Gluten (in hydrolysed foods)	Competitive ELISA	3 mg/kg	90	Biomedal Diagnostics
	Gluten-Tec® ELISA (5171GT)	Gliadin	Competitive ELISA	3.6 mg/kg	30	EuroProxima
Soybeans	RIDASCREEN FAST Soy	Soy protein	Sandwich ELISA	0.24 mg/kg	30	R-Biopharm AG
	RIDAQUICK Soy	Soya on Surfaces	Lateral Flow Strip	0.5 µg soya protein/100cm2	16	R-Biopharm AG

	VERATOX for Soy	Soy in processed foods, drinks and CIP	Sandwich ELISA	2.5 mg/kg	30	Neogen
	REVEAL 3-D for Soy	Soy	Lateral Flow Strip (with overflow line)	5 mg/kg; 2 μg/100cm2 swab	5	Neogen
	ALERT for Soy	Soy	Sandwich ELISA	2.5 mg/kg	30	Neogen
	Soy ESSOYPRD-48	Soy Flour Protein	Sandwich ELISA	2.5-25 mg/kg	80	ELISA Systems
	Soy ELISA Kit	Soy in raw & processed foods	Sandwich ELISA	0.31 mg/kg	<120	Crystal Chem
	AgraQuant® Soy	Soy	Sandwich ELISA	0.016 mg/kg	60	Romer Labs
	Monotrace Soy ELISA	Soy	Sandwich ELISA	LOD: 0.23 mg/kg LOQ: 1 mg/kg	40	BioFront Technologies
	Soja (Soy) ELISA	Soja trypsin inhibitor	Sandwich ELISA	0.016 mg/kg	60	Immunolab
	AlerTox Soy (STI)	Soja trypsin inhibitor	Sandwich ELISA	LOD: 0.016 mg/kg LOQ: 0.05 mg/kg	Not stated	Biomedal Diagnostics

	AlerTox Soy	Soy	Lateral Flow Strip	1 mg/kg	10	Biomedal Diagnostics
	PROTEON for Soy	Soy	Sandwich ELISA	LOD: 1.1 mg/kg LOQ: 3.5 mg/kg	90	Zeulab
	Soya Check	Soy	Sandwich ELISA	LOD: <0.7 mg/kg LOQ: 2 mg/kg	100	BioCheck (UK) Ltd
Milk	RIDASCREEN Fast Milk	Casein & B-lactoglobulin	Sandwich ELISA	0.7 mg/kg	30	R-Biopharm AG
	RIDASCREEN Fast Casein	Casein/caseinates	Sandwich ELISA	0.12 mg/kg casein in ice-cream/chocolate/beverages; 0.71 mg/kg casein for all other samples	30	R-Biopharm AG
	VERATOX for Casein	Casein	Sandwich ELISA	2.5 mg/kg	30	Neogen
	Casein ESCASPRD-48	Casein	Sandwich ELISA	1.0-10 mg/kg	45	ELISA Systems
	AgraQuant® Casein	Casein	Sandwich ELISA	0.04 mg/kg	60	Romer Labs
	Monotrace Milk	Casein	Sandwich	LOD: 0.12 mg/kg LOQ: 1 mg/kg	40	BioFront

	ELISA		ELISA			Technologies
	Casein ELISA Kit	Casein	Sandwich ELISA	0.31 mg/kg	Not stated	Crystal Chem
	Casein Lateral Flow Kit	Casein	Lateral Flow Strip	5 mg/kg	15	Crystal Chem
	BioKits RAPID 3D™ Casein Test	Casein	Lateral Flow Strip (with overflow line)	Screening test (no LOD)	5	Tepnel Reserach Products & Services
	AgraStrip® Casein	Casein	Lateral Flow Strip	2 mg/kg casein; 5 mg/kg skimmed milk powder	11	Romer Labs
	Casein ELISA	Casein	Sandwich ELISA	0.04 mg/kg	60	Immunolab
	AlerTox Casein ELISA	Casein	Sandwich ELISA	LOD: 0.05 mg/kg LOQ: 0.20 mg/kg	Not stated	Biomedal Diagnostics
	AlerTox Sticks Casein	Casein	Lateral Flow Strip	2.5 mg/kg	10	Biomedal Diagnostics
	Casein ELISA Kit	Casein	Sandwich ELISA	0.31 mg/kg	Not stated	Crystal Chem

	Milk-Check (Casein)	Casein	Sandwich ELISA	LOD: 0.2 mg/kg; LOQ: 0.8 mg/kg	100	BioCheck (UK) Ltd
	RIDASCREEN Fast B-lactoglobulin	B-lactoglobulin	Sandwich ELISA	0.19 mg/kg	30	R-Biopharm AG
	RIDASCREEN B-lactoglobulin	B-lactoglobulin in hydrolysed products	Competitive ELISA	0.1 mg/kg	165	R-Biopharm AG
	Beta Lactoglobulin ESMRDBLG-48	B-lactoglobulin	Sandwich ELISA	0.1-1.0 mg/kg	45	ELISA Systems
	Beta-Lactoglobulin ELISA Kit	B-lactoglobulin	Sandwich ELISA	0.31 mg/kg	Not Stated	Crystal Chem
	BioKits BLG Assay Kit	B-lactoglobulin	Sandwich ELISA	2 mg/kg	120	Neogen
	AgraQuant® B-lactoglobulin	B-lactoglobulin	Sandwich ELISA	0.0015 mg/kg	60	Romer Labs
	Beta-Lactoglobulin	B-lactoglobulin	Sandwich ELISA	0.0015 mg/kg	60	Immunolab

	ELISA					
	AlerTox BLG ELISA	B-lactoglobulin	Sandwich ELISA	LOD: 0.0015 mg/kg LOQ: 0.010 mg/kg	Not stated	Biomedal Diagnostics
	AlerTox Sticks BLG	B-lactoglobulin	Lateral Flow Strips	2.5 mg/kg	10	Biomedal Diagnostics
	Milk-Check (BLG)	B-lactoglobulin	Sandwich ELISA	LOD: <0.1 mg/kg LOQ: 0.2 mg/kg	100	BioCheck (UK) Ltd
	PROTEON Milk ELISA	B-lactoglobulin	Sandwich ELISA	LOD: 0.03 mg/kg LOQ: 0.05 mg/kg	90	Zeulab
	PROTEON EXPRESS for Milk	Milk	Lateral Flow Strip	1-2 mg/kg	10	Zeulab
	Bioavid Lateral Flow Milk	Milk	Lateral Flow Strip	1 mg/kg	8	R-Biopharm AG
	Milk Check	Milk	Sandwich ELISA	LOD: <0.1 mg/kg LOQ: 1 mg/kg	100	BioCheck (UK) Ltd
	VERATOX for Total Milk	Casein & Whey	Sandwich ELISA	2.5 mg/kg	30	Neogen
	REVEAL for Total	Casein & Whey	Lateral Flow	5 mg/kg	5	Neogen

	Milk		Strip			
	REVEAL 3-D for Total Milk	Casein & Whey	Lateral Flow Strip (with overflow line)	5 mg/kg	5	Neogen
	ALERT for Total Milk Allergen	Total Milk	Sandwich ELISA	5 mg/kg	30	Neogen
	Milk ELISA	Milk	Sandwich ELISA	0.005 mg/kg	60	Immunolab
	AlerTox Milk ELISA	Milk	Sandwich ELISA	LOD: 0.05 mg/kg LOQ: 0.5 mg/kg	Not stated	Biomedal Diagnostics
	Total Milk ESTMLK-48	Casein & Whey	Sandwich ELISA	0.25-2.5 mg/kg	45	ELISA Systems
Egg	RIDASCREEN FAST Egg Protein	Whole Egg Powder	Sandwich ELISA	0.1 mg/kg whole egg powder; 0.03 mg/kg egg white protein	30	R-Biopharm AG
	RIDASCREEN FAST Lysozym	Lysozyme	Sandwich ELISA	0.006 mg/kg lysozyme in wine; 0.016 mg/kg lysozyme in cheese + sausage	30	R-Biopharm AG
	Lysozyme ELISA	Lysozyme	Sandwich ELISA	0.002 mg/kg	60	Immunolab

	AlexTox Lysozyme ELISA	Lysozyme	Sandwich ELISA	LOD: 0.002 mg/kg LOQ: 0.025 mg/kg	Not stated	Biomedal Diagnostics
	VERATOX for Egg	Egg	Sandwich ELISA	2.5 mg/kg	30	Neogen
	REVEAL 3-D for Egg	Egg	Lateral Flow Strip (with overflow line)	5 mg/kg; 10 μg/100 cm2 egg swab	5	Neogen
	AlerTox Egg ELISA	Egg	Sandwich ELISA	LOD: 0.05 mg/kg LOQ: 0.4 mg/kg	Not stated	Biomedal Diagnostics
	AlerTox Sticks Egg	Egg	Lateral Flow Strip	1 mg/kg	10	Biomedal Diagnostics
	PROTEON Egg ELISA	Egg	Sandwich ELISA	LOD: 0.05 mg/kg LOQ: 0.18 mg/kg	90	Zeulab
	PROTEON EXPRESS for Egg	Egg	Lateral Flow Strip	1 mg/kg	10	Zeulab
	Egg Check	Egg	Sandwich ELISA	LOD: <0.2 mg/kg; LOQ: 0.4mg/kg	100	BioCheck (UK) Ltd
	BioKits RAPID	Ovomucoid (Gal d 1)	Lateral Flow	7.6 mg/kg egg white protein; 0.5 mg/kg	5	Tepnel

	3D™ Egg Test		Strip (with overflow line)	whole egg powder; 5 µg/25cm2 whole egg powder environmental swab		Reserach Products & Services
	Enhanced Egg Residue Kit	Ovomucoid	Sandwich ELISA	1.0-10 mg/kg	Not Stated	ELISA Systems
	Monotrace Egg ELISA	Ovomucoid	Sandwich ELISA	LOD: 0.3 mg/kg LOQ: 1 mg/kg	40	BioFront Technologies
	Egg (Ovalbumin) ELISA Kit	Ovalbumin	Sandwich ELISA	0.31 mg/kg	Not Stated	Crystal Chem
	Egg (Ovalbumin) Lateral Flow Kit	Ovalbumin	Lateral Flow Strip	5 mg/kg	15	Crystal Chem
	BioKits Egg Assay Kit	Ovalbumin	Sandwich ELISA	0.1 mg/kg	75	Neogen
	Ovalbumin ELISA	Ovalbumin	Sandwich ELISA	0.004 mg/kg	60	Immunolab
	AlerTox Ovalbumin ELISA	Ovalbumin	Sandwich ELISA	LOD: 0.004 mg/kg LOQ: 0.025 mg/kg	Not stated	Biomedal Diagnostics
	Egg White ELISA	Egg White	Sandwich ELISA	0.05 mg/kg	60	Immunolab

	AgraQuant® Egg White	Egg White	Sandwich ELISA	0.05 mg/kg	60	Romer Labs
	AgraStrip® Egg	Egg (Dried Whole Egg)	Lateral Flow Strip	2 mg/kg	11	Romer Labs
Nuts	RIDASCREEN FAST Mandel/Almond	Almond	Sandwich ELISA	1.2 mg/kg	30	R-Biopharm AG
	Bioavid Lateral Flow Almond	Almond	Lateral Flow Strip	1 mg/kg	10	R-Biopharm AG
	VERATOX for Almond	Almond	Sandwich ELISA	LOD: 0.3 mg/kg LOQ:2.5 mg/kg	30	Neogen
	REVEAL 3-D for Almond	Almond	Lateral Flow Strip (with overflow line)	5 mg/kg almond; 1 µg/100cm3 almond protein	5	Neogen
	Almonds ESARD-48	Almond	Sandwich ELISA	0.5-5 mg/kg	50	ELISA Systems
	AgraQuant® Almond	Almond	Sandwich ELISA	0.2 mg/kg	60	Romer Labs
	AgraStrip®	Almond	Lateral Flow	2 mg/kg	11	Romer Labs

	Almond		Strip			
	Almond ELISA	Almond	Sandwich ELISA	0.2 mg/kg	60	Immunolab
	Monotrace Almond ELISA	Almond	Sandwich ELISA	LOD: 0.15 mg/kg LOQ: 1 mg/kg	40	BioFront Technologies
	AlerTox Almond ELISA	Almond	Sandwich ELISA	LOD: 0.2 mg/kg LOD: 0.5 mg/kg	Not stated	Biomedal Diagnostics
	AlerTox Sticks Almond	Almond	Lateral Flow Strip	10 mg/kg	10	Biomedal Diagnostics
	Almond Check	Almond	Sandwich ELISA	LOD: <0.25 mg/kg LOQ: 0.5 mg/kg	100	BioCheck (UK) Ltd
	Bioavid Lateral Flow Brazil Nut	Brazil Nut	Lateral Flow Strip	1 mg/kg	10	R-Biopharm AG
	AgraStrip® Brazil Nut	Brazil Nut	Lateral Flow Strip	5 mg/kg	11	Romer Labs
	Brazil nut ELISA	Brazil Nut	Sandwich ELISA	0.2 mg/kg	60	Immunolab
	Monotrace Brazil Nut ELISA	Brazil Nut	Sandwich ELISA	LOD: 0.14 mg/kg LOQ: 1 mg/kg	40	BioFront Technologies

	RIDASCREEN FAST Cashew	Cashew	Sandwich ELISA	0.09 mg/kg	30	R-Biopharm AG
	Bioavid Lateral Flow Cashew Kernel	Cashew Kernel	Lateral Flow Strip	1 mg/kg	10	R-Biopharm AG
	Cashew ELISA	Cashew (4% cross reactivity with pistachio)	Sandwich ELISA	0.2 mg/kg	60	Immunolab
	Monotrace Cashew ELISA	Cashew	Sandwich ELISA	LOD: 0.12 mg/kg LOQ: 1 mg/kg	40	BioFront Technologies
	AlerTox Cashew ELISA	Cashew	Sandwich ELISA	LOD: 0.2 mg/kg LOD: 2 mg/kg	Not stated	Biomedal Diagnostics
	Cashew Check	Cashew	Sandwich ELISA	LOD: <0.2 mg/kg; LOQ: 2 mg/kg	100	BioCheck (UK) Ltd
	Bioavid Lateral Flow Coconut	Coconut	Lateral Flow Strip	1 mg/kg	10	R-Biopharm AG
	Coconut ELISA	Coconut	Sandwich ELISA	0.4 mg/kg	60	Immunolab
	Monotrace Coconut ELISA	Coconut	Sandwich ELISA	0.13 mg/kg	40	BioFront Technologies

	AletTox Coconut	Coconut	Sandwich ELISA	LOD: 0.4 mg/kg LOQ: 2 mg/kg	Not stated	Biomedal Diagnostics
	RIDASCREEN FAST Hazelnut	Hazelnut	Sandwich ELISA	1.5 mg/kg	30	R-Biopharm AG
	Bioavid Lateral Flow Hazelnut	Hazelnut	Lateral Flow Strip	1 mg/kg	10	R-Biopharm AG
	VERATOX for Hazelnut	Hazelnut	Sandwich ELISA	2.5 mg/kg	30	Neogen
	REVEAL 3-D for Hazelnut	Hazelnut	Lateral Flow Strip (with overflow line)	5-10 mg/kg; 10 µg/100cm2 swab	5	Neogen
	Hazelnut ESHRD-48	Hazelnut	Sandwich ELISA	0.5-5 mg/kg	35	ELISA Systems
	AgraQuant® Hazelnut	Hazelnut	Sandwich ELISA	0.3 mg/kg	60	Romer Labs
	AgraStrip® Hazelnut	Hazelnut	Lateral Flow Strip	5 mg/kg	11	Romer Labs
	Hazelnut ELISA	Hazelnut	Sandwich ELISA	0.3 mg/kg	60	Immunolab

	Monotrace Hazelnut ELISA	Hazelnut	Sandwich ELISA	0.04 mg/kg	40	BioFront Technologies
	AlerTox Hazelunut ELISA	Hazelnut	Sandwich ELISA	LOD:0.3 mg/kg; LOQ: 1 mg/kg	Not stated	Biomedal Diagnostics
	AleTox Sticks Hazelnut	Hazelnut	Lateral Flow Strip	1 mg/kg	10	Biomedal Diagnostics
	Hazelnut Check	Hazelnut	Sandwich ELISA	LOD:<0.5 mg/kg LOQ: 1 mg/kg	100	BioCheck (UK) Ltd
	RIDASCREEN FAST Macademia	Macademia	Sandwich ELISA	0.38 mg/kg	30	R-Biopharm AG
	Bioavid Lateral Flow Macademia Nut	Macademia	Lateral Flow Strip	1 mg/kg	10	R-Biopharm AG
	AgraStrip® Macademia Nut	Macademia	Lateral Flow Strip	2 mg/kg	11	Romer Labs
	Macademia Nut ELISA	Macademia	Sandwich ELISA	0.1 mg/kg	60	Immunolab
	Monotrace	Macademia	Sandwich	0.13 mg/kg	40	BioFront

	Macademia Nut ELISA		ELISA			Technologies
	AlerTox Macademia ELISA	Macademia	Sandwich ELISA	LOD: 0.1 mg/kg LOQ: 1 mg/kg	Not stated	Biomedal Diagnostics
	RIDASCREEN FAST Peanut	Peanut	Sandwich ELISA	1.3 mg/kg	30	R-Biopharm AG
	Bioavid Lateral Flow Peanut	Peanut	Lateral Flow Strip	≤1 mg/kg	10	R-Biopharm AG
	VERATOX for Peanut	Peanut	Sandwich ELISA	2.5 mg/kg	30	Neogen
	REVEAL for Peanut	Peanut	Lateral Flow Strip	5 mg/kg	10	Neogen
	REVEAL 3-D for Peanut	Peanut	Lateral Flow Strip (with overflow line)	5 mg/kg; 1 µg/100cm2 swab	5	Neogen
	Peanut ESPRDT-48	Peanut (Ara h1 & Ara h2)	Sandwich ELISA	1-15 mg/kg	35	ELISA Systems
	Peanut Lateral	Peanut	Lateral Flow	5 mg/kg	15	Crystal Chem

	Flow Kit		Strip			
	Peanut ELISA Kit	Peanut	Sandwich ELISA	0.31 mg/kg	Not Stated	Crystal Chem
	BioKits Peanut Assay Kit	Peanut	Sandwich ELISA	0.1 mg/kg	75	Neogen
	ALERT for Peanut	Peanut	Sandwich ELISA	5 mg/kg	30	Neogen
	AgraQuant® Peanut	Peanut	Sandwich ELISA	0.1 mg/kg	60	Romer Labs
	AgraStrip® Peanut	Peanut	Lateral Flow Strip	1 mg/kg	11	Romer Labs
	Peanut ELISA	Peanut	Sandwich ELISA	0.1 mg/kg	60	Immunolab
	Monotrace Peanut ELISA	Peanut	Sandwich ELISA	LOD: 0.24 mg/kg LOQ: 1 mg/kg	40	BioFront Technologies
	AlerTox Peanut ELISA	Peanut	Sandwich ELISA	LOD: 0.3 mg/kg LOQ: 1 mg/kg	Not stated	Biomedal Diagnostics
	AleTox Sticks Peanut	Peanut	Lateral Flow Strip	1 mg/kg	10	Biomedal Diagnostics

	Peanut Check	Peanut	Sandwich ELISA	LOD: <0.5 mg/kg LOQ: 1 mg/kg	100	BioCheck (UK) Ltd
	Pistachio Check	Pistachio	Sandwich ELISA	LOD: <0.2 mg/kg LOQ: 1 mg/kg	100	BioCheck (UK) Ltd
	Pistachio ELISA	Pistachio	Sandwich ELISA	0.13 mg/kg	60	Immunolab
	Bioavid Lateral Flow Pistachio	Pistachio	Lateral Flow Strip	1 mg/kg	10	R-Biopharm AG
	Monotrace Pistachio ELISA	Pistachio	Sandwich ELISA	LOD: 0.12 mg/kg LOQ: 1 mg/kg	40	BioFront Technologies
	AlerTox Pistachio ELISA	Pistachio	Sandwich ELISA	LOD: 0.13 mg/kg LOQ: 1 mg/kg	Not stated	Biomedal Diagnostics
	AgraStrip® Cashew/Pistachio	Cashew/Pistachio	Lateral Flow Strip	5 mg/kg	11	Romer Labs
	Monotrace Pine Nut ELISA	Pine Nut	Sandwich ELISA	LOD: 0.24 mg/kg LOQ: 1 mg/kg	40	BioFront Technologies
	Monotrace Pecan ELISA	Pecan	Sandwich ELISA	LOD: 0.17 mg/kg; LOQ: 1 mg/kg	40	BioFront Technologies
	Pecan nut ELISA	Pecan	Sandwich	0.2 mg/kg	60	Immunolab

			ELISA			
	Bioavid Lateral Flow Walnut	Walnut & Pecan	Lateral Flow Strip	10 mg/kg	10	R-Biopharm AG
	BioKits Walnut Assay	Walnut	Sandwich ELISA	0.25 mg/kg	75	Neogen
	AgraQuant® Walnut	Walnut	Sandwich ELISA	0.35 mg/kg	60	Romer Labs
	AgraStrip® Walnut	Walnut	Lateral Flow Strip	10 mg/kg	11	Romer Labs
	Walnut ELISA	Walnut	Sandwich ELISA	0.35 mg/kg	60	Immunolab
	Monotrace Walnut ELISA	Walnut	Sandwich ELISA	LOD:0.22 mg/kg LOQ: 1 mg/kg	Not stated	BioFront Technologies
	AlerTox Walnut ELISA	Walnut	Sandwich ELISA	LOD: 0.6 mg/kg LOQ: 2 mg/kg	Not stated	Biomedal Diagnostics
	Walnut Check	Walnut	Sandwich ELISA	LOD: <1.0 mg/kg LOQ: 2 mg/kg	100	BioCheck (UK) Ltd
	REVEAL for Multi-Treenut	Almond, cashew, hazelnut, walnut, pecan & pistachio	Lateral Flow Strip	Rinse samples: 5-10 mg/kg; CIP swabs: 20 μg/100cm2	10	Neogen

Oil Plants	RIDASCREEN FAST Sesame	Sesame	Sandwich ELISA	0.2 mg/kg	30	R-Biopharm AG
	Bioavid Lateral Flow Sesame	Sesame	Lateral Flow Strip	1 mg/kg	10	R-Biopharm AG
	REVEAL 3-D for Sesame	Sesame	Lateral Flow Strip (with overflow line)	5 mg/kg	5	Neogen
	Sesame ESSESRD-48	Sesame	Sandwich ELISA	0.5-5 mg/kg	65	ELISA Systems
	BioKits Sesame Assay Kit	Sesame	Sandwich ELISA	1 mg/kg	180	Neogen
	AgraQuant® Sesame	Sesame	Sandwich ELISA	0.2 mg/kg	60	Romer Labs
	AgraStrip® Sesame	Sesame	Lateral Flow Strip	5 mg/kg	11	Romer Labs
	Sesame ELISA	Sesame	Sandwich ELISA	0.2 mg/kg	60	Immunolab
	Monotrace Sesame ELISA	Sesame	Sandwich ELISA	0.3 mg/kg	40	BioFront Technologies

	AleTox Sesame ELISA	Sesame	Sandwich ELISA	LOD: 0.2 mg/kg LOD: 2 mg/kg	Not stated	Biomedal Diagnostics
	Sesame Check	Sesame	Sandwich ELISA	LOD: <0.2 mg/kg LOQ: 2 mg/kg	100	BioCheck (UK) Ltd
Seafood	RIDASCREEN FAST Crustacean	Tropomyosin	Sandwich ELISA	2 mg/kg	30	R-Biopharm AG
	Bioavid Lateral Flow Crustacean	Tropomyosin	Lateral Flow Strip	10 mg/kg	10	R-Biopharm AG
	VERATOX for Crustacea	Tropomyosin	Sandwich ELISA	2.5 mg/kg	30	Neogen
	REVEAL 3-D for Crustacea	Tropomyosin	Lateral Flow Strip (with overflow line)	5-10 mg/kg; 40 µg/100cm2 cooked prawn extract	5	Neogen
	Crustacean ESCRURD-48	Tropomyosin	Sandwich ELISA	0.05-0.50 mg/kg	60	ELISA Systems
	AgraQuant® Crustacea	Tropomyosin	Sandwich ELISA	0.09 mg/kg	60	Romer Labs
	Crustacean ELISA	Tropomyosin	Sandwich ELISA	Soy sauce: 0.0017 mg/kg; vegetable soup: 0.0036 mg/kg; bakery products:	60	Immunolab

				0.0009 mg/kg; fish: 0.0085 mg/kg; meat: 0.0103 mg/kg		
	Monotrace Crustacea ELISA	Tropomyosin	Sandwich ELISA	LOD: 0.07 mg/kg LOQ: 1mg/kg	40	BioFront Technologies
	AlerTox Crustacean ELISA	Tropomyosin	Sandwich ELISA	LOD: 0.001 mg/kg LOQ: 0.020 mg/kg	Not stated	Biomedal Diagnostics
	AlerTox Sticks Crustacea	Tropomyosin	Lateral Flow Strips	7 mg/kg (dry) 33 mg/kg (wet)	10	Biomedal Diagnostics
	Crustacea-Check	Tropomyosin	Sandwich ELISA	LOD: 0.1 mg/kg LOQ: 1.2 mg/kg	100	BioCheck (UK) Ltd
	Fish Check	Fish	Sandwich ELISA	LOD: <1.0 mg/kg LOQ: 5 mg/kg	100	BioCheck (UK) Ltd
	AlerTox Sticks Fish	Fish	Lateral Flow Strips	0.35 mg/kg (dry) 1 mg/kg (wet)	10	Biomedal Diagnostics
	AlerTox Fish ELISA	Fish	Sandwich ELISA	LOD: 1.4 mg/kg LOQ 4 mg/kg	Not stated	Biomedal Diagnostics
	Fish ELISA	Fish	Sandwich ELISA	1.4 mg/kg	60	Immunolab

Miscellanous	RIDASCREEN FAST Lupine	Lupin	Sandwich ELISA	0.7 mg/kg	30	R-Biopharm AG
	VERATOX for Lupine	Lupin	Sandwich ELISA	2.5 mg/kg	30	Neogen
	Lupin ESLFP-48	Lupin Flour Protein	Sandwich ELISA	0.5-5 mg/kg	Not Stated	ELISA Systems
	AgraQuant® Lupin	Lupin	Sandwich ELISA	0.2 mg/kg	60	Romer Labs
	Lupine ELISA	Lupin	Sandwich ELISA	Sausage: 0.2 mg/kg; bread: 0.3 mg/kg; orange juice: 0.7 mg/kg; ketchup: 0.1 mg/kg; croquette: 0.2 mg/kg	60	Immunolab
	Monotrace Lupin ELISA	Lupin	Sandwich ELISA	LOD: 0.16 mg/kg LOQ: 1 mg/kg	40	BioFront Technologies
	AlerTox Lupine ELISA	Lupin	Sandwich ELISA	LOD: 0.2 mg/kg LOD: 2 mg/kg	Not stated	Biomedal Diagnostics
	Lupin Check	Lupin	Sandwich ELISA	LOD: <0.3 mg/kg LOQ: 2 mg/kg	100	BioCheck (UK) Ltd
	RIDASCREEN FAST Mustard	Mustard (white, yellow, brown & black)	Sandwich ELISA	0.22 mg/kg	30	R-Biopharm AG

	Bioavid Lateral Flow Mustard	Mustard	Lateral Flow Strip	1 mg/kg	10	R-Biopharm AG
	VERATOX for Mustard	Mustard	Sandwich ELISA	2.5-25 mg/kg	30	Neogen
	REVEAL 3-D for Mustard	Mustard	Lateral Flow Strip (with overflow line)	5 mg/kg; 2 μg/100cm2 swab	5	Neogen
	Mustard ESMUS-48	Mustard	Sandwich ELISA	1-10 mg/kg	Not Stated	ELISA Systems
	AgraQuant® Mustard	Mustard	Sandwich ELISA	1 mg/kg	60	Romer Labs
	AgraStrip® Mustard	Mustard	Lateral Flow Strip	2 mg/kg	11	Romer Labs
	Mustard ELISA	Mustard	Sandwich ELISA	1 mg/kg	60	Immunolab
	Monotrace Mustard ELISA	Mustard	Sandwich ELISA	LOD: 0.13 mg/kg LOQ: 1 mg/kg	40	BioFront Technologies
	AlerTox Mustard ELISA	Mustard	Sandwich ELISA	LOD: 1 mg/kg LOQ: 2 mg/kg	Not stated	Biomedal Diagnostics

	Mustard Check	Mustard	Sandwich ELISA	LOD: <1 mg/kg LOQ: 2 mg/kg	100	BioCheck (UK) Ltd

Table S4 Conventionally lab-based allergen detection methods and their smartphone-based counterparts

**Conventional Method**	**Current Method**	**Summary**
ELISA with microplate reader as detector system	ELISA with smartphone-based microplate reader as detector system	ELISA is a traditionally laboratory based routine allergen detection method, as it requires multiple reagent handling steps, sample extraction and detection from a UV-VIS microplate spectrophotometer. Microplate readers are expensive and non-portable, however using a smartphone as the microplate reader (see section 3.2.1) for detecting the colorimetric reaction makes the method more portable and accessible in low-resource settings.
Lateral flow immunoassay (LFIA) with strip reader	LFIA with smartphone reader	LFIA is a rapid, portable, easy to use, safe and disposable screening method applied in food allergen analysis. LFIA qualitative optical detection is based on the appearance of 1 or 2 lines for absence/presence of allergens. To quantify LFIA results, conventionally a strip test reader is required which is a relatively expensive piece of equipment requiring training/instruction for use and a power source to operate. By using a smartphone as a LFIA reader (see section 3.1.2) quantitative/semi-quantitative results are achievable. Smartphones have the added benefits of being portable, affordable, easy to use and can connect wirelessly to deliver instant results to shareholders.
Flow cytometry	Miniaturised flow cytometry	Flow cytometry is a lab based immunoassay where antibodies of interest are immobilised onto different coloured microbeads, it has the benefit of being able to

		be easily multiplexed. The flow cytometer is a desk based instrument. Miniaturised flow cytometry (MFC) reduces the flow channels in the cytometer to microfluidic channels, reducing the overall size of the instrument. MFC is capable of being linked with a smartphone as the readout system (see section 3.3.1), this gives the method the ability to be portable and suitable for on-site detection of allergens, and data processing and results to be disseminated through a customised MFC app.
Surface plasmon resonance (SPR)	Smartphone based SPR	SPR is a traditionally lab based method which can detect changes in antibody/antigen binding on the gold surface of a sensor chip. SPR is able to detect multiple allergens, simultaneously in real time and monitor their binding responses in the form of a sensorgram. Smartphone based SPR can be achieved through a 3D printed optical attachment, the phone camera and a PDMS prism for light to be deflected from. By linking with a smartphone, SPR becomes a portable method which requires no analyte labelling, limited sample preparation steps and results which can be seen in real time.

References

1. Raz SL, H. Norde, W. Bremer, MGEG (2010) Food Allergen Profiling with an Image Plasmon Resonance-Based Biosensor Anal Chem 82 (20):8485-8491

2. Badran AA, Morais S, Maquieira Á (2017) Simultaneous determination of four food allergens using compact disc immunoassaying technology. Anal Bioanal Chem 409 (9):2261-2268. doi:10.1007/s00216-016-0170-0

3. Blais BW, Gaudreault M, Phillippe LM (2003) Multiplex enzyme immunoassay system for the simultaneous detection of multiple allergens in foods. Food Control 14 (1):43-47. doi:https://doi.org/10.1016/S0956-7135(02)00053-1

4. Weng X, Gaur G, Neethirajan S (2016) Rapid Detection of Food Allergens by Microfluidics ELISA-Based Optical Sensor. Biosensors 6 (2):24. doi:10.3390/bios6020024

5. Yman IM, Eriksson A, Johansson MA, Hellens K-E (2006) Food Allergen Detection with Biosensor Immunoassays. J AOAC Int 89 (3):856-861

6. Gomaa A, Boye J (2015) Simultaneous detection of multi-allergens in an incurred food matrix using ELISA, multiplex flow cytometry and liquid chromatography mass spectrometry (LC–MS). Food Chem 175:585-592. doi:https://doi.org/10.1016/j.foodchem.2014.12.017

7. Otto G, Lamote A, Deckers E, Dumont V, Delahaut P, Scippo M-L, Pleck J, Hillairet C, Gillard N (2016) A flow-cytometry-based method for detecting simultaneously five allergens in a complex food matrix. J Food Sci Technol 53 (12):4179-4186. doi:10.1007/s13197-016-2402-x

8. Cho CY, Nowatzke W, Oliver K, Garber EAE (2015) Multiplex detection of food allergens and gluten. Anal Bioanal Chem 407 (14):4195-4206. doi:10.1007/s00216-015-8645-y

9. Gomaa AR, S. Boye, J (2012) Detection of allergens in a multiple allergen matrix and study of the impact of thermal processing Journal of Nutrition & Food Sciences 9:1-6

10. Wang W, Li Y, Zhao F, Chen Y, Ge Y (2011) Optical thin-film biochips for multiplex detection of eight allergens in food. Food Res Int 44 (10):3229-3234. doi:https://doi.org/10.1016/j.foodres.2011.08.013

11. Tortajada-Genaro LS-F, S. Morais, S. Gabaldon. Puchades, R. Maquiera, A. (2012) Multiplex DNA detection of food allergens on a Digital Versatile Disk. J Agric Food Chem 60:36-43

12. Cheng F, Wu J, Zhang J, Pan A, Quan S, Zhang D, Kim H, Li X, Zhou S, Yang L (2016) Development and inter-laboratory transfer of a decaplex polymerase chain reaction assay combined with capillary electrophoresis for the simultaneous detection of ten food allergens. Food Chem 199:799-808. doi:https://doi.org/10.1016/j.foodchem.2015.12.058

13. Luber F, Demmel A, Pankofer K, Busch U, Engel K-H (2015) Simultaneous quantification of the food allergens soy bean, celery, white mustard and brown mustard via combination of tetraplex real-time PCR and standard addition. Food Control 47:246-253. doi:https://doi.org/10.1016/j.foodcont.2014.06.047

14. Waiblinger H-U, Boernsen B, Geppert C, Demmel A, Peterseil V, Koeppel R (2017) Ring trial validation of single and multiplex real-time PCR methods for the detection and quantification of the allergenic food ingredients mustard, celery, soy, wheat and rye. J Verbrauch Lebensm 12 (1):55-72. doi:10.1007/s00003-016-1063-z

15. Waiblinger H-UB, B. Naumann, G. Koeppel, R. (2014) Ring trial validation of single and multiplex real-time PCR methods for the detection and quantification of the allergenic food ingredients sesame, almond, lupine and Brazil nut. J Verbrauch Lebensm 9:297-310

16. Wu JC, L. Lin, D. Ma, Zhaocheng. Deng, X. (2016) Development and Application of a Multiplex Real Time PCR Assay as an Indicator of Potential Allergenicity in Citrus Fruits. J Agric Food Chem 64 (47):9089-9098

17. Pafundo S, Gullì M, Marmiroli N (2010) Multiplex real-time PCR using SYBR® GreenER™ for the detection of DNA allergens in food. Anal Bioanal Chem 396 (5):1831-1839. doi:10.1007/s00216-009-3419-z

18. Hubalkova Z, Rencova E (2011) One-step multiplex PCR method for the determination of pecan and Brazil nut allergens in food products. J Sci Food Agric 91 (13):2407-2411. doi:10.1002/jsfa.4479

19. López-Calleja IM, García A, Madrid R, García T, Martín R, González I (2017) Multiplex ligation-dependent probe amplification (MLPA) for simultaneous detection of DNA from sunflower, poppy, flaxseed, sesame and soy allergenic ingredients in commercial food products. Food Control 71:301-310. doi:https://doi.org/10.1016/j.foodcont.2016.06.014

20. Mustorp SD, SM. Holck, AL. (2011) Multiplex, Quantitative, Ligation-Dependent Probe Amplification for Determination of Allergens in Food. J Agric Food Chem 59:5231-5239

21. Ehlert AD, A. Hupfer, C. Busch, U. Engel, KH. (2009) Simultaneous detection of DNA from 10 food allergens by ligation-dependent probe amplification. Food Additives & Contaminants: Part A 26 (4):409-418. doi:10.1080/02652030802593529

22. Unterberger C, Luber F, Demmel A, Grünwald K, Huber I, Engel K-H, Busch U (2014) Simultaneous detection of allergenic fish, cephalopods and shellfish in food by multiplex ligation-dependent probe amplification. Eur Food Res Technol 239 (4):559-566. doi:10.1007/s00217-014-2251-7

23. Ng E, Nadeau KC, Wang SX (2016) Giant magnetoresistive sensor array for sensitive and specific multiplexed food allergen detection. Biosens Bioelectron 80:359-365. doi:https://doi.org/10.1016/j.bios.2016.02.002
